# Pyrazoline derivatives as promising novel antischistosomal agents

**DOI:** 10.1038/s41598-021-02792-0

**Published:** 2021-12-06

**Authors:** Cristiane S. Morais, Ana C. Mengarda, Fábio B. Miguel, Karine B. Enes, Vinícius C. Rodrigues, Maria Cristina C. Espírito-Santo, Abolghasem Siyadatpanah, Polrat Wilairatana, Mara R. C. Couri, Josué de Moraes

**Affiliations:** 1 Research Center for Neglected Diseases, Guarulhos University, Praça Tereza Cristina, 229, Centro, Guarulhos, SP 07023-070 Brazil; 2 Department of Chemistry, Federal University of Juiz de Fora, Juiz de Fora, MG 36036-900 Brazil; 3 Laboratory of Immunopathology of Schistosomiasis (LIM-06), Department of Infectious and Parasitic Diseases, Faculty of Medicine, University of São Paulo, São Paulo, SP Brazil; 4 Laboratory of Helminthology, Institute of Tropical Medicine, University of São Paulo, São Paulo, SP Brazil; 5 Ferdows School of Paramedical and Health, Birjand University of Medical Sciences, 9717853577 Birjand, Iran; 6 Department of Clinical Tropical Medicine, Faculty of Tropical Medicine, Mahidol University, Bangkok, 10400 Thailand

**Keywords:** Drug discovery, Microbiology, Antimicrobials, Antiparasitic agents

## Abstract

Praziquantel is the only available drug to treat schistosomiasis, a parasitic disease that currently infects more than 240 million people globally. Due to increasing concerns about resistance and inadequate efficacy there is a need for new therapeutics. In this study, a series of 17 pyrazolines (**15–31**) and three pyrazoles (**32–34**) were synthesized and evaluated for their antiparasitic properties against ex vivo adult *Schistosoma mansoni* worms. Of the 20 compounds tested, six had a 50% effective concentration (EC_50_) below 30 μM. Our best hit, pyrazoline **22**, showed promising activity against adult schistosomes, with an EC_50_ < 10 µM. Additionally, compound **22** had low cytotoxicity, with selectivity index of 21.6 and 32.2 for monkey and human cell lines, respectively. All active pyrazolines demonstrated a negative effect on schistosome fecundity, with a marked reduction in the number of eggs. Structure–activity relationship analysis showed that the presence of the non-aromatic heterocycle and N-substitution are fundamental to the antischistosomal properties. Pharmacokinetics, drug-likeness and medicinal chemistry friendliness studies were performed, and predicted values demonstrated an excellent drug-likeness profile for pyrazolines as well as an adherence to major pharmaceutical companies’ filters. Collectively, this study demonstrates that pyrazoline derivatives are promising scaffolds in the discovery of novel antischistosomal agents.

## Introduction

Schistosomiasis is an infectious disease which leads to significant economic and public health consequences, particularly in the poorest communities. It is a water-associated disease caused by infection with parasitic trematodes of the genus *Schistosoma*^1^. With an estimated global prevalence of 240 million infected people, schistosomiasis is intimately correlated with poverty, and the disease is associated with a chronic and debilitating morbidity manifested by sequelae such as cognitive impairment, growth stunting, and decreased physical fitness, among other pathological effects^2,3^. Disease pathology is due to immunologic reactions to *Schistosoma* eggs trapped in tissues, mainly in the liver and spleen^1,4^. Among species that infect humans, *Schistosoma mansoni* has the widest geographical distribution, being found in Africa, the Middle East, South America and the Caribbean^3^.

The global schistosomiasis control strategy relies upon preventive chemotherapy with praziquantel, via mass drug administration. Estimates show that at least 236.6 million people required preventive treatment with praziquantel in 2019^3^. Although the drug is effective against all schistosome species, numerous persistent schistosomiasis hotspots remain^5–7^. In addition, low cure rates and concern with drug resistance have recently been reported^8^. Praziquantel has also been extensively used for the control of platyhelminth parasites in domestic and livestock animals, and populations of animals with flatworm infections that were not eliminated despite multiple treatments with praziquantel have been identified^9^. Since treatment options for flatworm infections are limited in both human and veterinary medicine, there is an urgent need to identify novel antischistosomal agents as an alternative treatment for schistosomiasis and other flatworm infections^10,11^.

In recent years, a significant number of nitrogen heterocycles have been approved by the Food and Drug Administration (FDA) as chemotherapeutic drugs^12,13^. In the search for new antischistosomal agents, studies have been directed towards investigating the schistosomicidal activities of synthetic heterocyclic compounds e.g.^14,15^. Pyrazole, and its reduced form pyrazoline, are the electron-rich nitrogen heterocycles which play an important role in diverse biological activities^16^. These compounds are privileged scaffolds in medicinal chemistry, and various works have been reported enumerating the antiparasitic potential of pyrazoles and pyrazolines. For example, a series of pyrazole–pyrazoline substituted with benzenesulfonamide were synthesized and evaluated for their antimalarial activity in vitro and in vivo^17^. More recently, pyrazol(in)e derivatives of curcumin analogs have been reported as a new class of trypanosomicidal agents^18^. However, the reports on schistosomicidal (or anthelmintic) activities of pyrazoles and pyrazolines are scarce. In this study, a series of 17 pyrazoline derivatives were synthesized and tested in vitro on *S. mansoni* adult worms. Compounds were thereafter tested on mammalian cells to determine the selectivity of schistosomicidal compounds. In addition, a structure–activity relationship (SAR) study to determine the effects of the different functional groups present in these molecules was undertaken, and three pyrazoles derivatives were also tested in vitro against adult schistosomes. Finally, physicochemical properties, pharmacokinetics, drug-likeness and medicinal chemistry friendliness analysis were performed with all active compounds against *S. mansoni*.

## Results and discussion

### Chemistry

**Figure 1:**
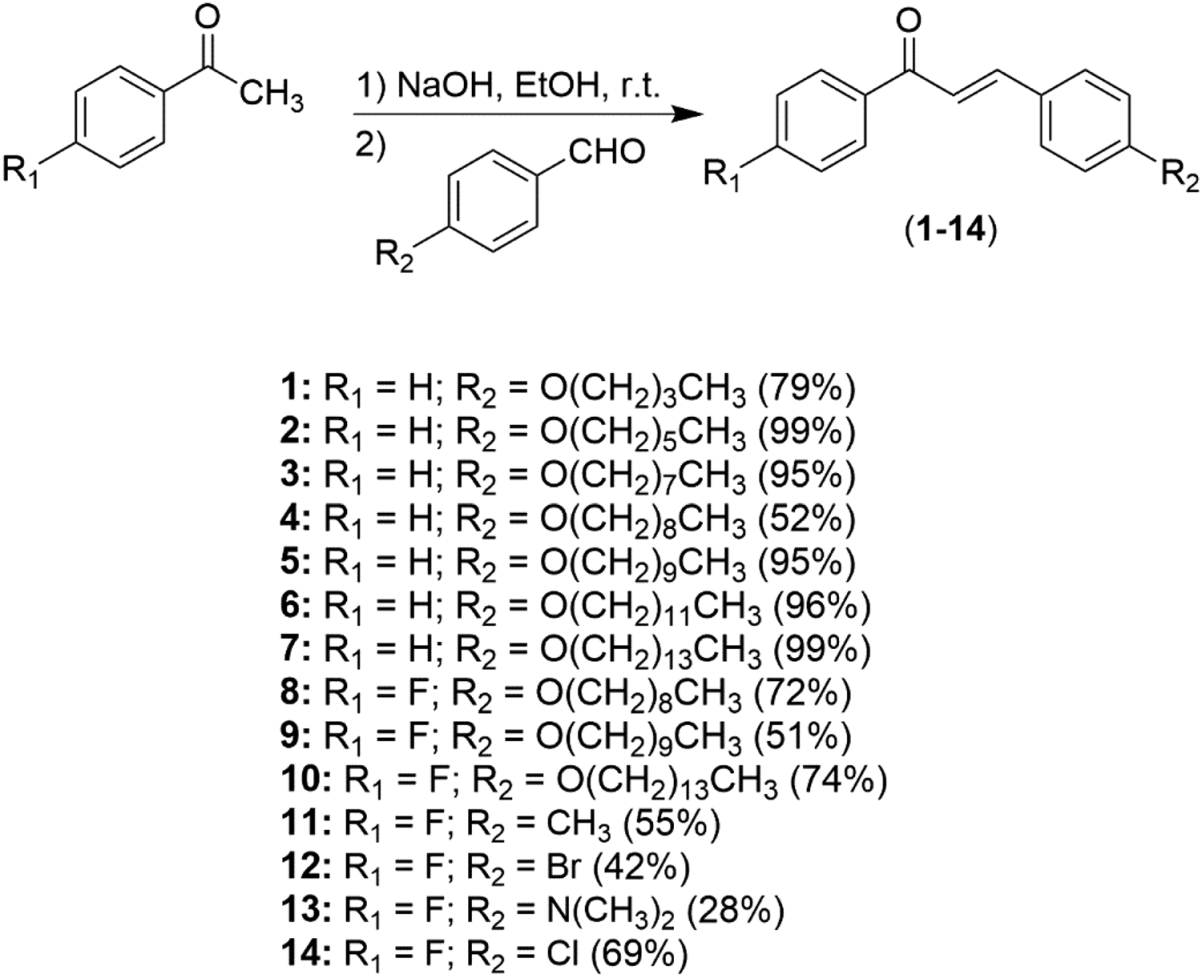
General synthesis of chalcone derivatives.

**Figure 2:**
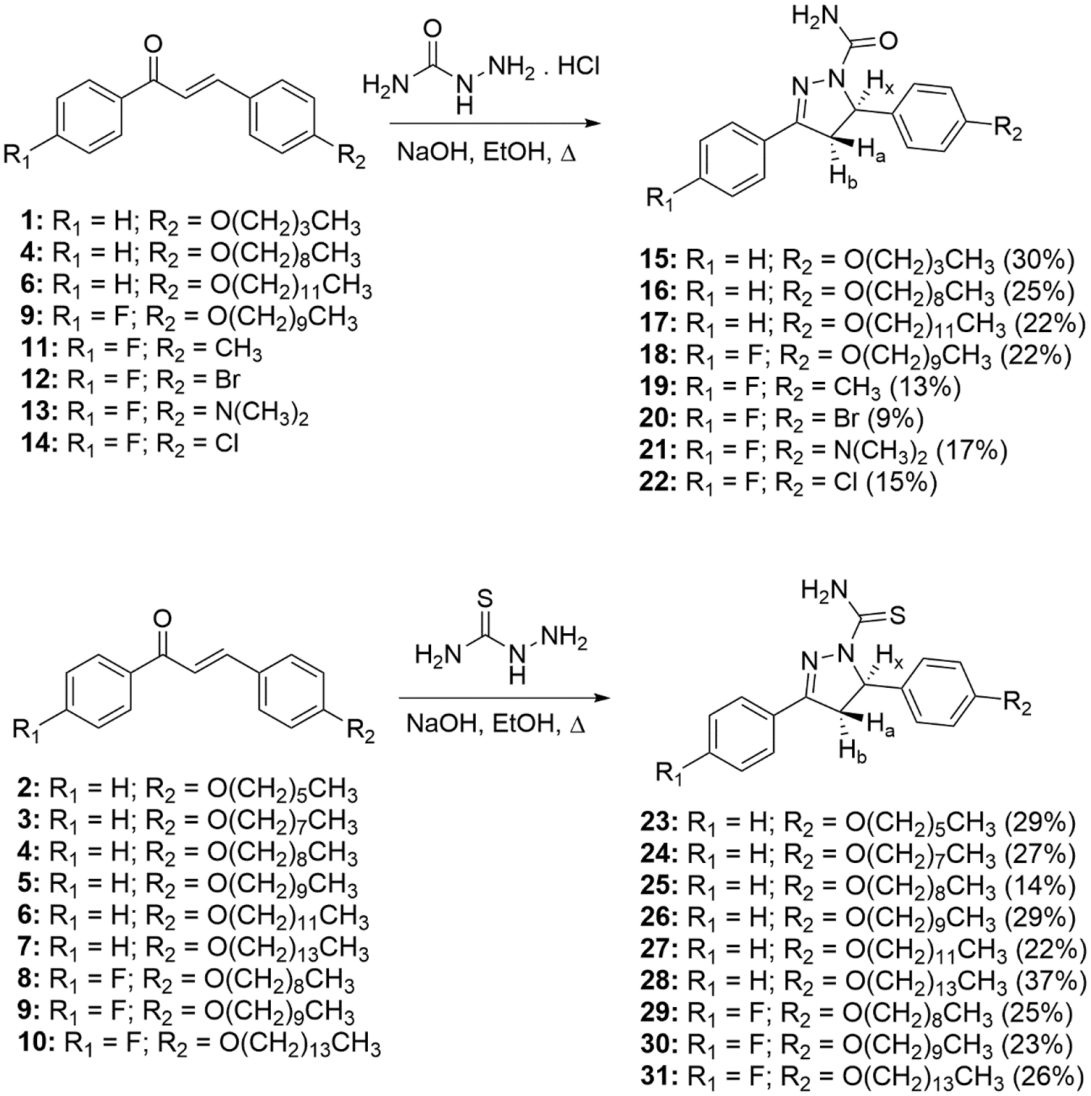
General synthesis of pyrazolines derivatives.

**Figure 3:**
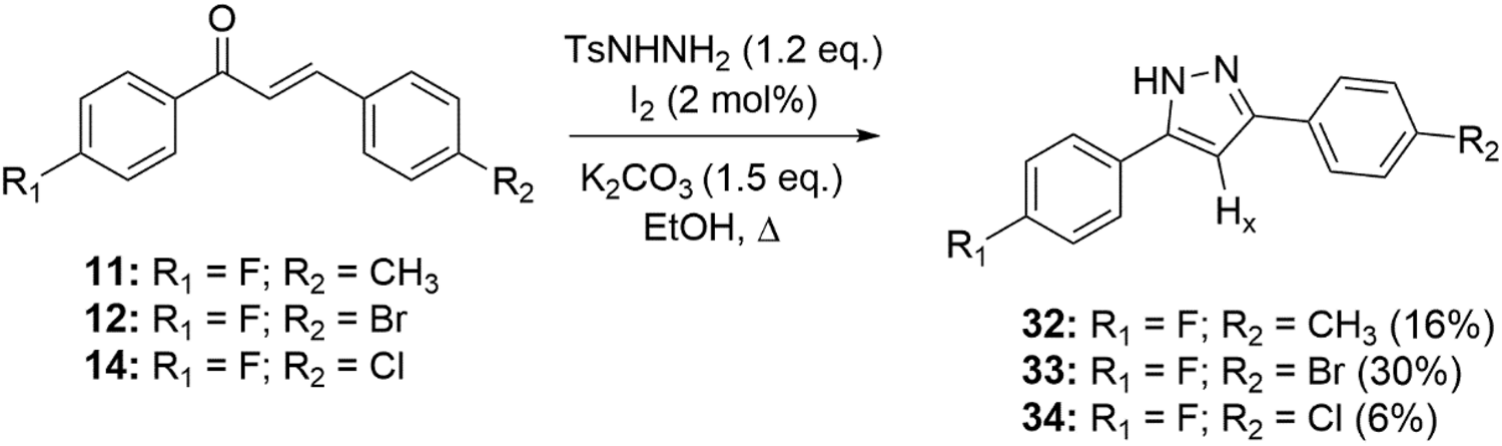
General synthesis of pyrazoles derivatives.

The synthesis of the pyrazoles and pyrazolines was initiated by the reaction between acetophenone and aromatic aldehyde in basic solution, generating the appropriate chalcone in 28–99% yield (Fig. 1). The latter was treated with semicarbazide hydrochloride or thiosemicarbazide, leading to the formation of the desired pyrazoline compound 15–31 (9–37% yield) (Fig. 2). Furthermore, chalcones **11**, **12** and **14** were reacted with TsNHNH2 and iodine, leading to pyrazoles 32–34 in 6–30% yields (Fig. 3).

### Anthelmintic activity against *S. mansoni*

For biological screening, *S. mansoni* adult parasites were isolated from the mouse infection model (parasite ex vivo) and all compounds were initially screened at 50 µM. The gold-standard antiparasitic compound praziquantel was used as a positive control and DMSO at 0.5% v/v (representing the highest concentration of solvent) served as a negative control. Of the 17 pyrazolines tested, six (**16, 18, 19, 21, 22** and **23**) showed antiparasitic activity after 72 h, and these compounds were further tested at a range of concentrations for their EC_50_ determination. Results of the EC_50_ value for each tested drug are summarized in Table 1. Among the six pyrazolines selected, compound **22** was the most active against adult schistosomes, with a EC_50_ value of 6.2 μM. Pyrazolines **16, 18, 19** and **21** also displayed high antischistosomal properties (EC_50_ < 20 μM), whereas **23** showed moderate anthelmintic activity (EC_50_ of 28.2 μM). Using the log rank (Mantel–Cox) test, comparison of antiparasitic activity revealed that the order of potency was **22** (*P* < 0.001), **16** and **18** (*P* < 0.01), **19** and **21** (*P* < 0.01), and **23** (*P* < 0.001). For the remaining compounds tested, no EC_50_ values could be calculated due to lack of activity at 50 μM. The positive control drug praziquantel was confirmed to be highly active, with a EC_50_ of 0.93 μM. In tandem, our findings confirm that pyrazolines have a potent antiparasitic activity. Indeed, few compounds in the literature are active at low concentrations for review see^11,19^. According to criteria established by the World Health Organization (WHO) for antiparasitic hits, leads, and drug candidates, at the start of a screening campaign a compound should inhibit mobility of helminths in vitro at 10 μg/mL^20^, commonly ranging from 30 to 40 μM. Thus, the anthelminthic activity of pyrazolines surpasses criteria established by the WHO.

**Table 1:** In vitro activity of pyrazolines against *S. mansoni* adult worm and and cytotoxicity.

Compounds	*S. mansoni*	Monkey cells		Human cells	
	EC_50_ (μM)	CC_50_ (μM)	SI	CC_50_ (μM)	SI
**15**	> 50	N.D	N.D	N.D	N.D
**16**	13.8 [9.6–17.4]*	154.8 [122.5–184.2]	11.2	> 200	> 14.5
**17**	> 50	N.D	N.D	N.D	N.D
**18**	14.6 [9.4–19.1]	158.9 [118.9–194.3]	10.9	> 200	> 13.7
**19**	17.4 [11.2–21.6]	168.6 [120.6–200]	9.7	> 200	> 11.5
**20**	> 50	N.D	N.D	N.D	N.D
**21**	19.8 [12.4–23.9]	> 200	> 10.1	> 200	> 10.1
**22**	6.2 [4.1–10.3]	134.3 [101.2–160.6]	21.6	> 200	> 32.2
**23**	28.2 [21.7–35.9]	> 200	> 7.1	> 200	> 7.1
**24**	> 50	N.D	N.D	N.D	N.D
**25**	> 50	N.D	N.D	N.D	N.D
**26**	> 50	N.D	N.D	N.D	N.D
**27**	> 50	N.D	N.D	N.D	N.D
**28**	> 50	N.D	N.D	N.D	N.D
**29**	> 50	N.D	N.D	N.D	N.D
**30**	> 50	N.D	N.D	N.D	N.D
**31**	> 50	N.D	N.D	N.D	N.D
**32**	> 50	N.D	N.D	N.D	N.D
**33**	> 50	N.D	N.D	N.D	N.D
**34**	> 50	N.D	N.D	N.D	N.D
**PZQ**	0.93 [0.81–1.2]	> 200	> 200	> 200	> 200

*EC*_*50*_ effetive concentration 50% against adult schistosomes, *CC*_*50*_ cytotoxic concentration 50% against monkey (Vero) or human (SH-SY5Y) cells, *SI* selectivity index, *ND* not determined.

*95% Confidence Interval.

**Figure 4:**
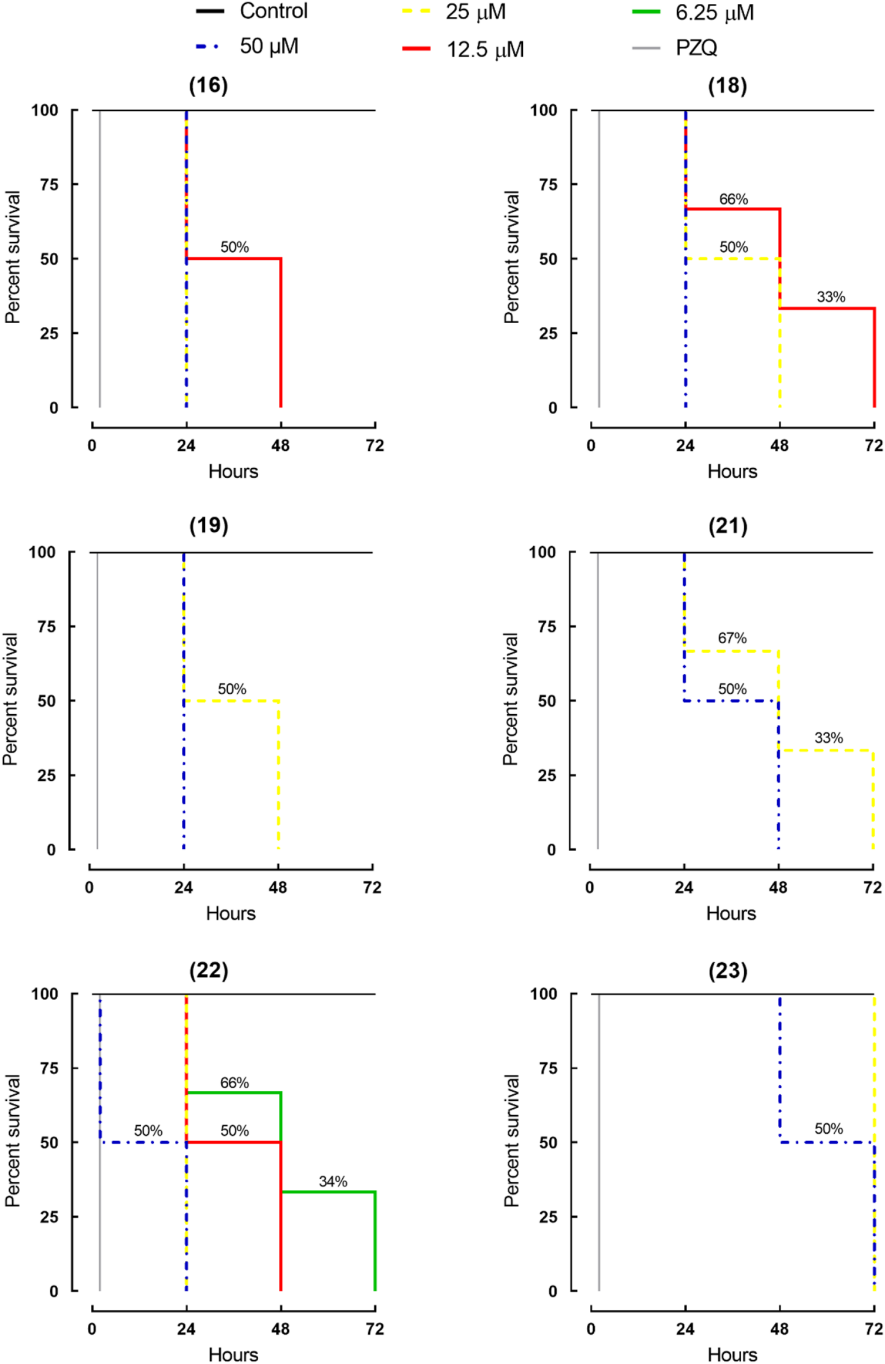
Viability of ex vivo adult *S. mansoni* worms following exposure to pyrazolines **16**, **18**, **19**, **21**, **22** and **23**. Parasites were obtained from mice by perfusion 49 days after infection. Parasites were monitored for up to 72 h, and results are expressed as the percent mortality recorded by Kaplan–Meier survival curves. Mean values of viability were derived from a minimum of three experiments (n = 3). Control: drug-free medium. PZQ: praziquantel at 2 µM.

Assays regarding the survival times of *S. mansoni* were also performed with pyrazolines **16**, **18**, **19**, **21**, **22** and **23** to understand the kinetics and mode of action of these compounds. The viability of the schistosomes over a period of 72 h in vitro is demonstrated in Fig. 4. Control worms remained viable over the entire observation period. Pyrazolines induced mortality in schistosomes in a time- and concentration-dependent manner. In more details, pyrazolines **16, 18, 19** and **22** were able to kill all parasites within 24 h of contact at a concentration of 50 μM. Under the same timetable, the death of all worms exposed to compounds **16** and **22** at a concentration of 25 μM was observed. A slightly slower onset of action was seen when schistosomes were incubated with pyrazolines **21** and **23**. For example, all parasites died after 48 h of exposure to compound **21** at 50 μM, whereas compound **23** at 50 μM induced 100% mortality after 72 h. The precise mechanism of action, however, remains to be determined. The known antischistosomal drug praziquantel shows a fast-killing profile against adult *S. mansoni*.

**Figure 5:**
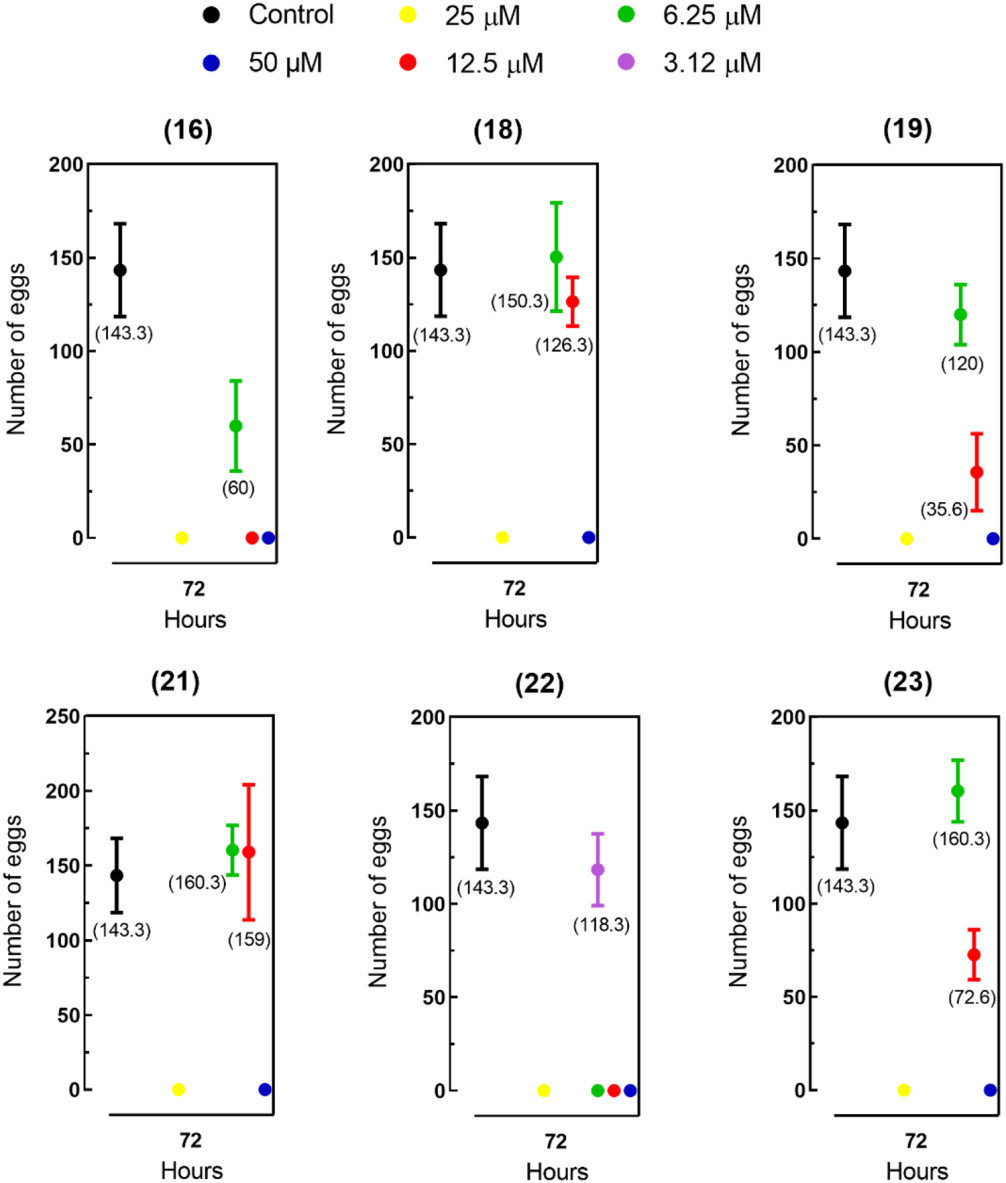
Number of eggs released by paired adult schistosomes exposed to pyrazolines **16**, **18**, **19**, **21**, **22** and **23**. Control: drug-free medium. Data are presented as the mean ± SD from three experiments (n = 3).

Based on antischistosomal properties of pyrazolines **16, 18, 19, 21, 22** and **23**, the ability of these six compounds to affect fecundity (egg production) was subsequently measured. Interestingly, a complete lack of oviposition was observed when adult worm pairs were exposed to pyrazolines at 25 and 50 μM. To further investigate this effect on schistosome fecundity, parasites were tested using sublethal concentrations (3.5 μM for compound **22**, 6.25 μM for compound **16**, and 12.5 μM for compounds **18, 19, 21**, and **23**). Compared to control parasites, pyrazolines **16, 19**, and **23** demonstrated a negative effect on schistosome fecundity, with a reduction of 30–70% in the number of eggs (Fig. 5). Due to the high relevance of eggs for both disease transmission and pathology, the results revealed the potential of pyrazolines **16, 18, 19, 21, 22** and **23**, as antischistosomal agents.

### Cytotoxicity evaluation and selectivity index

The selectivity index (SI) of a compound is a widely accepted parameter used to express a drug’s in vitro efficacy. Therefore, the cytotoxic effect of these six pyrazolines (**16, 18, 19, 21, 22** and **23**) was evaluated using a monkey cell line (Vero) and a human cell line (SH-SY5Y). Cells were incubated with each compound at a range of concentrations for determination of cytotoxic concentrations 50% (CC_50_). The average CC_50_ of each pyrazoline is summarized in Table 1. All pyrazolines active against *S. mansoni* showed a low potential of cytotoxicity, with CC_50_ values > 130 μM and > 200 μM for Vero and SH-SY5Y, respectively. With the EC_50_ data collected for schistosomes as well as the CC_50_ for animal and human cells, the selectivity indices (SI) were determined for each of the pyrazolines tested (Table 1). Pyrazoline **22** had the lowest CC_50_ values (134.3 μM for Vero and > 200 μM for SH-SY5Y); however, it also had the lowest EC_50_ values of all the pyrazolines tested (6.2 μM), which resulted in the higher SI scores (21.6 and 32.2 for Vero and SH-SY5Y, respectively). Compound **16**, with an EC_50_ = 13.8 μM and a CC_50_ = 154.8 μM (Vero) and a CC_50_ = 200 μM (SH-SY5Y), resulted in a SI = 11.2 and a SI = 14.5, for animal and human cell lines, respectively. Similar data for EC_50_ and CC_50_ were recorded for compounds **18** and **19**, resulting in SI values of 10.9 and 9.7 for Vero cells and 13.7 and 11.5 for SH-SY5Y, respectively. Compound **21**, with an EC_50_ = 19.8 μM and cytotoxicity greater than 200 μM, resulted in SI > 10 for both animal and human cell lines. A SI value ≥ 10 is in accordance with the criteria defined by the WHO for antiparasitic hits, leads, and drug candidates^20^. In this study, most pyrazolines tested had SI value ≥ 10, demonstrating the highly selective anthelmintic effect of pyrazolines. Comparatively, the SI values achieved with pyrazolines exceeded those obtained for other relevant heterocyclic compounds with antischistosomal properties^21,22^. Although the SI values recorded for pyrazolines were more than 10 times below those for the reference drug praziquantel, a SI value ≥ 10 fulfills and exceeds the criteria established by the WHO for potential compounds as anthelmintic agents^20^.

### SAR plan and analysis

The According to these biological results, it is possible to establish a structural relationship among the compounds tested. Of the 17 pyrazolines tested, 13 have *O*-alkyl chains in their structures (**15–18**, **23–31**), but only three of these compounds (**16, 18** and **23**), with carbon chains of different sizes (9, 10 and 6C, respectively), showed some biological potential. These results may indicate that this change is not an essential factor for the biological activity of this series of compounds.

In agreement with the previous inference, four (**19–22**) compounds without *O*-alkyl chains in their structure were tested, and three of them (**19, 21** and **22**) showed good schistosomicidal activity, which indicates that the presence of less bulky groups as substituents in the aromatic rings favors the biological activity of the compounds^23^.

Among the eight compounds derived from the semicarbazide tested (**15–22**), five (**16, 18, 19, 21** and **22**) had biological potential with EC_50_ between 6.2 and 19.8 µM and selectivity ranging from 9.7 to 21.6 and four (**18, 19, 21** and **22**) of the compounds had the fluorine substituent in the para position of the aromatic ring from acetophenone, which suggests that halogen plays an important role in the antiparasitic activity of such compounds^23^.

Regarding compounds derived from thiosemicarbazide, among the nine compounds tested (**23–31**), only compound 23 showed antischistosomal activity with EC_50_ = 28.2 µM and IS > 7.

Of the six compounds (**16, 18, 19, 21, 22** and **23**) that showed activity against *S. mansoni*, five (**16, 18, 19, 21** and **22**) are derived from semicarbazide, while only compound **23** is derived from thiosemicarbazide. This may indicate that the presence of the semicarbazide portion is of relative importance for the antischistosomal activity of pyrazolines.

In order to locate the pharmacophoric group of the compounds tested, the anti*s*chistosomal properties of some pyrazoles, aromatic nitrogenous heterocycles of the same class as pyrazolines, were also evaluated. Regarding this scenario, the influence of the pyrazoline heterocycle and the N-substitution of the compounds on their biological activity was evaluated. Thus, three pyrazoles (**32–34**) were tested under the same conditions. None of the three pyrazoles showed biological activity against *S. mansoni* adult worms. This may indicate that the presence of the non-aromatic heterocycle and N-substitution are fundamental to the antischistosomal properties of the compounds, as observed while comparing pyrazoles **32** and **34**, which do not have biological potential (EC_50_ > 50 µM), and pyrazolines **19** and **22**, which have good activity with EC_50_ of 17.4 and 6.2 µM and selectivity index of 9.7 and 21.6, respectively.

### Physicochemical properties and drug-likeness parameters

**Figure 6:**
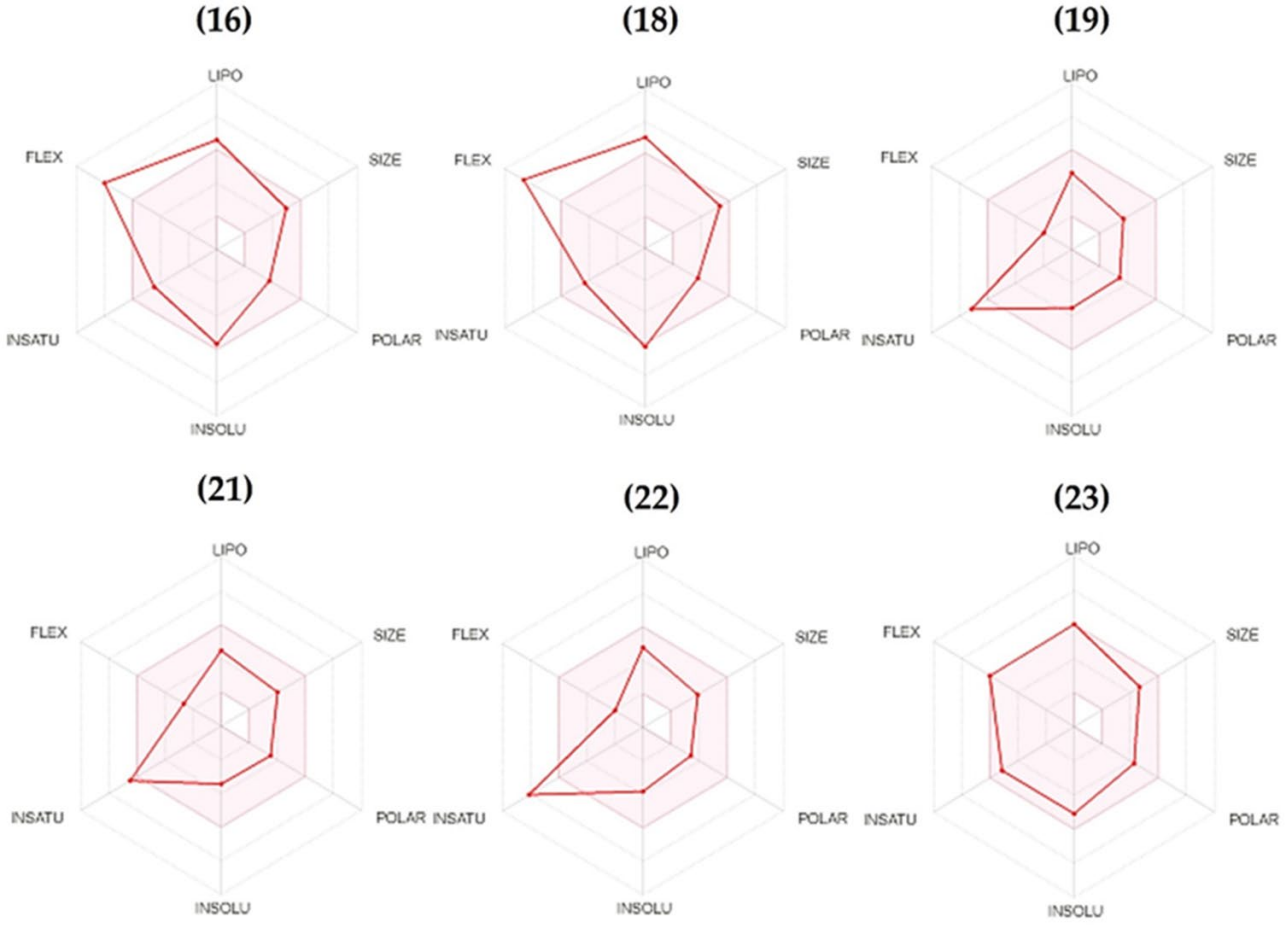
Bioavailability radar plots of pyrazolines **16**, **18**, **19**, **21**, **22** and **23**. The pink area represent the optimal range for each property, and the red line represents the values of the six calculated properties. lipophilicity: XLOGP3 between − 0.7 and + 5.0, size: molecular weight between 150 and 500 g/mol, polarity: topological polar surface area (TPSA) between 20 and 130 Å^2^, solubility: log S not higher than 6, saturation: fraction of carbons in the sp3 hybridization not less than 0.25, and flexibility: no more than 9 rotatable bonds. Plots were performed using the SwissADME tool.

The druggability of pyrazolines **16, 18, 19, 21, 22** and **23** was performed in silico to investigate the physicochemical properties, pharmacokinetic parameters, and drug-likeness (Fig. 6, Tables 2 and 3). Initially, the obtained results on the bioavailability radar (Fig. 6), which considers six physicochemical properties such as lipophilicity, size, polarity, solubility, flexibility and saturation to detect drug-likeness, indicate good adherence of pyrazolines to all evaluated parameters, except flexibility (compounds **16** and **18**) and unsaturation (compounds **19** and **22**).

**Table 2:** Physicochemical and pharmacokinetic prediction for pyrazolines **16**, **18**, **19**, **21**, **22** and **23**.

Parameters	Pyrazolines					
	**16**	**18**	**19**	**21**	**22**	**23**
ClogP	4.12	4.7	3.3	2.97	3.57	3.51
TPSA (Å^2^)	67.92	67.92	58.69	61.93	58.69	82.94
GI absorption	High	High	High	High	High	High
BBB permeation	Yes	No	Yes	Yes	Yes	No
CYP1A2 inhibitor	No	No	No	No	No	No
P-gp substrate	Yes	No	No	No	No	No
PAINS	No	No	No	Yes	No	No
Brenk alert	No	No	No	No	No	Yes
SA	4.04	4.18	3.29	3.39	3.21	3.90

*ClogP* logarithm of n-octanol/water, *TPSA* topological polar surface area, *GI* gastrointestinal absorption, *BBB* blood–brain barrier penetration, *CYP1A2* cytochrome P450 family 1 subfamily A member 2, involved in the metabolism of xenobiotics, *PAINS* pan-assay interference substructures, *SA* Synthetic accessibility, scored from 1 (very easy) to 10 (very difficult). In silico prediction was performed using SwissADME platform.

**Table 3:** Drug-likeness and adherence to major pharmaceutical companies’ filters.

Pharmaceutical companies	Pyrazolines					
	**16**	**18**	**19**	**21**	**22**	**23**
Pfizer (Lipinski)^a^	Yes, 0 violation	Yes, 0 violation	Yes, 0 violation	Yes, 0 violation	Yes, 0 violation	Yes, 0 violation
GSK (Veber)^b^	No, 1 violation: Rotors > 10	No, 1 violation: Rotors > 10	Yes, 0 violation	Yes, 0 violation	Yes, 0 violation	Yes, 0 violation
Pharmacia (Egan)^c^	Yes	No, 1 violation: WLOGP > 5.88	Yes, 0 violation	Yes, 0 violation	Yes, 0 violation	Yes, 0 violation
Bayern (Muegge)^d^	No, 1 violation: XLOGP3 > 5	No, 1 violation: XLOGP3 > 5	Yes, 0 violation	Yes, 0 violation	Yes, 0 violation	Yes, 0 violation
Amgen (Ghose)^e^	No, 1 violation: MR > 130	No, 2 violations: WLOGP > 5.6, MR > 130	Yes, 0 violation	Yes, 0 violation	Yes, 0 violation	Yes, 0 violation

*MW* molecular weight, *MR* molecular refractivity, *LogP* logarithm of n-octanol/water, *XLOGP3* atomistic method including corrective factors and knowledge-based library, *WLOGP* atomistic method based on the fragmental system, *HBA* hydrogen bond acceptor, *HBD* hydrogen bond donor, *TPSA* topological polar surface area. In silico prediction was performed using SwissADME platform.

^a^Lipinski filter^24^: MW ≤ 500; LogP ≤ 5; HBA ≤ 10; HBD ≤ 5.

^b^Veber filter^25^: Rotatable bonds ≤ 10; TPSA ≤ 140.

^c^Egan filter^26^: WLOGP ≤ 5.88; TPSA ≤ 131.6.

^d^Muegge filter^27^: 200 ≤ MW ≤ 600; − 2 ≤ XLOGP ≤ 5; TPSA ≤ 150; rings ≤ 7; carbon > 4; heteroatoms > 1; rotatable bonds ≤ 15; HBA ≤ 10; HBD ≤ 5.

^e^Ghose filter^44^: 160 ≤ MW ≤ 480; -0.4 ≤ WLOGP ≤ 5.6; − 0.4 ≤ MR ≤ 130; 20 ≤ atoms ≤ 70.

Our in silico analysis also suggested adequate “drug-like” parameters associated with gastrointestinal absorption (GI) and oral bioavailability, according to ClogP and TPSA values. With the exception of compound 16, pharmacokinetic analysis also indicated no interactions with P-glycoprotein (P-gp). Pyrazolines **16, 19, 21**, and **22** were predicted to be permeant to the blood brain barrier (BBB), a possibly interesting feature in cases of cerebral schistosomiasis. Moreover, the prediction for these compounds did not indicate inhibitory activity for the CYP1A2 isoforms, emphasizing the low potential for drug interactions. Computational analysis also suggested that pyrazolines **16, 18, 19, 22** and **23** are not Pan-Assay Interference Sub-structures (PAINS, also kwon as frequent hitters or promiscuous compounds)^28^. Thus, there is a reduced probability that the antischistosomal properties of the tested pyrazolines could be considered artifacts caused by promiscuous reactivity. In addition, with the exception of compound **23**, which had a thiocarbonyl group, no structural alert identified from Brenk filter for drug discovery for neglected diseases^29^ was identified for pyrazolines. Collectively, these findings suggested that pyrazolines exhibited desirable characteristic of a drug candidates.

In the pharmaceutical industry, there has been a growing interest in the computational prediction of synthetic difficulty^30^. All six active pyrazolines are synthetically viable and accessible to experimental testing, as demonstrated by computational prediction of a synthetically accessibility score. It is important to report that all compounds were easily synthetized in sufficient quantities for in vitro bioassays.

The adherence to leading pharmaceutical companies such as Pfizer (Lipinski)^31^, GSK (Veber)^32^, Pharmacia (Egan)^33^, Bayer (Muegge)^34^ and Amgen (Ghose)^35^ by computational filters was also performed to evaluate the drug-likeness of pyrazolines (Table 3). Interestingly, none of the tested pyrazolines violated Lipinski’s rule, the most widely used filter to estimate solubility and permeability in drug discovery and development settings. Considering the filters developed by major pharmaceutical companies, pyrazolines **19, 21, 22** and **23** followed five drug-likeness rules, supporting the use of these compounds as suitable drug candidates.

In conclusion, a series of 17 pyrazolines (**15–31**) and three pyrazoles (**32–34**) were synthesized and six (**16, 18, 19, 21, 22** and **23**) were found to have an antiparasitic activity in the micromolar range when tested against *S. mansoni *ex vivo. These compounds have low cytotoxicity for animal and human cell lines, with an excellent selectivity profile. In addition, all active pyrazolines demonstrated a negative effect on schistosome fecundity, with a marked reduction in the number of eggs. Structure–activity relationship analysis showed that the presence of the non-aromatic heterocycle and N-substitution are fundamental to the antischistosomal properties. As recommended for drug screening against helminths, a promising agent should be active against adult worms at 10 μg/mL (typically ranging from 30 to 40 μM) and display a high SI (≥ 10). In this study, all these requirements were fulfilled, especially by **16, 18, 21**, and **22**. Finally, physicochemical properties and drug-likeness parameters of pyrazolines demonstrated excellent adherence to all analyzed properties as well as in adherence to major pharmaceutical companies. Among the six pyrazolines tested, compound **22** was highly active against adult schistosomes (EC_50_ value of 6.2 μM) and it displayed desirable characteristic of a good drug candidate. Overall, this study demonstrates that pyrazoline derivatives are promising scaffolds in the discovery of new antischistosomal agents and opens new avenues in the search for drug candidates against schistosomiasis.

## Methods

### Chemistry

Chalcones, pyrazolines and pyrazoles were prepared according to the method previously described^36,37^. The chemical structures of all compounds are presented in Figs. 1, 2 and 3.

All the reagents and solvents were purchased as reagent grade and used without any purification. Thin layer chromatography (TLC) was performed on glass plates (silica gel F254; Merck), using ultraviolet light (264 nm) and/or iodine vapor as revelator. Melting points were determined on a MQAPF-Microquimica apparatus. IR spectra were acquired using a Bruker Alpha-E ATR (Attenuated Total Reflection) spectrometer. NMR data were recorded on a Bruker 500 Advance Spectrometer. NMR experiments were conducted in deuterochloroform (CDCl3) and deuterodimethylsulfoxide (DMSO-d6). The chemical shifts values (δ) were reported in parts per million (ppm) with tetramethylsilane (TMS) as internal reference. The following abbreviations are used to explain the multiplicities: s = singlet; d = doublet; dd = double doublet; t = triplet and brs = broad singlet and coupling constants (J) are reported in Hertz (Hz).

All the structural data collected are presented in Supplementary Information.

### Preparation of chalcones derivatives (1–14)

Into an ethanolic solution of NaOH 4 M (1.6 g of NaOH in 10 mL of ethanol) was added 1 equivalent of the respective acetophenone (4 mmol) and 1 equivalent of the aromatic aldehyde (4 mmol). The reaction was left under magnetic stirring at room temperature until conversion was complete, as evidenced by TLC (eluent: 100% DCM, revelator: ultraviolet light and I2 vapor). The crude was neutralized with 4 M HCl and kept in refrigerator overnight. Once precipitated, they were vacuum filtered and washed with ethanol. The crude was recrystallized from ethanol or methanol in order to obtain the pure compounds. The assignment of the structures is fully supported by their characteristic shift values.

### Preparation of pyrazolines derivatives (15–31)

The pyrazolines were synthesized according to a previously published procedure^22^. A solution of the appropriate chalcone (1 eq.) in absolute ethanol (20 mL) and hydrazide (semicarbazide hydrochloride or thiosemicarbazide, 2 eq.) was treated with NaOH (2.5 eq.) in ethanol (10 mL). The reaction mixture was heated under reflux until the complete disappearance of the starting materials as evidenced by TLC (revelator: ultraviolet light, iodine vapors and/or 20% H_2_SO_4_ solution). The precipitates were filtered, washed with a cold mixture of ethanol/water, dried and recrystallized from suitable solvent to give pure compound. The 1H NMR spectra of compounds **15–31** are shown in Supplementary Information.

*5-(4-butoxyphenyl)-4,5-dihydro-3-phenylpyrazole-1-carboxamide (****15****).* Yield: 30%; 1H NMR (300 MHz, DMSO-d6) δ (ppm): 0.90 (t, 3H, J = 6.6 Hz, CH_3_); 1.34–1.70 (m, 4 H, –CH_2_–); 3.03 (dd, 1H, Ja,x = 5.1 Hz, Ja,b = 17.7 Hz, Ha); 3.76 (dd, 3H, Jb,x = 12.0 Hz, Jb,a = 17.7 Hz, Hb); 3.91 (t, 2H, J = 6.6 Hz, –OCH2–); 5.35 (dd, 1H, Jx,a = 5.1 Hz, Jx,b = 12.0 Hz, Hx); 6.49 (sl, 2H, NH); 6.84–7.80 (m, 9H).

*4,5-dihydro-5-(4-(nonyloxy)phenyl)-3-phenylpyrazole-1-carboxamide (****16****).* Yield: 25%; mp: 91.8–94.2 °C; 1H NMR (300 MHz, CDCl3) δ (ppm): 0.86 (sl, 3H, CH3); 1.25–1.77 (m, 14 H, –CH2–); 3.14 (dd, 1H, Ja,x = 4.8 Hz, Ja,b = 17.7 Hz, Ha); 3.73 (dd, 1H, Jb,x = 12.0 Hz, Jb,a = 17.7 Hz, Hb); 3.88 (t, 2H, J = 6.6 Hz, –OCH2–5.46 (dd, 1H, Jx,a = 4.8 Hz, Jx,b = 12.0 Hz, Hx); 6.84–7.71 (m, 9H). 13C NMR (75 MHz, CDCl3) δ (ppm): 14.2–32.0 (Caliphatic); 43.1; 59.8; 68.2 (–OCH2–); 115.0–134.6 (Caromatic); 152.1 (C=N); 155.4 (C=O); 158.8.

*5-(4-(dodecyloxy)phenyl)-4,5-dihydro-3-phenylpyrazole-1-carboxamide (****17****).* Yield: 22%; mp: 94.8–97.9 °C; 1H NMR (300 MHz, DMSO-d6) δ (ppm): 0.84 (t, 3H, J = 6.6 Hz, CH3); 1.22–1.70 (m, 20 H, –CH2–); 3.02 (dd, 1H, Ja,x = 5.1 Hz, Ja,b = 18.0 Hz, Ha); 3.76 (dd, 1H, Jb,x = 12.0 Hz, Jb,a = 18.0 Hz, Hb); 3.89 (t, 2H, J = 6.6 Hz, –OCH2–); 5.35 (dd, 1H, Jx,a = 5.1 Hz, Jx,b = 12.0 Hz, Hx); 6.47 (sl, 2H, NH), 6.82–7.79 (m, 9H). 13C NMR (75 MHz, DMSO-d6) δ (ppm): 14.3–31.7 (Caliphatic); 42.7; 59.6; 67.8 (–OCH2–); 114.8–135.9 (Caromatic); 150.9 (C=N); 155.3 (C=O); 158.1.

*5-(4-(decyloxy)phenyl)-3-(4-fluorophenyl)-4,5-dihydropyrazole-1-carboxamide (****18****).* Yield: 22%; mp: 109.2–113.0 °C; 1H NMR (300 MHz, DMSO-d¬6) δ (ppm): 0.82 (t, 3H, J = 6.0 Hz, CH3); 1.21–1.67 (m, 16 H, –CH2–); 3.00 (dd, 1H, Ja,x = 5.1 Hz, Ja,b = 17.7 Hz, Ha); 3.70–3.89 (m, 3H, Hb, CH2); 5.35 (dd, 1H, Jx,a = 5.1 Hz, Jx,b = 12.0 Hz, Hx); 6.50 (sl, 2H, NH); 6.81–7.84 (m, 8H). 13C NMR (75 MHz, DMSO-d6) δ (ppm): 13.9–31.3 (Caliphatic); 42.3; 59.3; 67.4 (–OCH2–); 114,3–135.4 (Caromatic); 149.6 (C=N); 154.9 (C=O); 157.7; 162.9 (d, 1 J = 246.0 Hz).

*3-(4-fluorophenyl)-4,5-dihydro-5-p-tolylpyrazole-1-carboxamide (****19****).* Yield: 13%; mp: 151.0–153.0 °C; 1H NMR (300 MHz, DMSO-d6) δ (ppm): 2.25 (s, 3H, CH3); 3.02 (dd, 1H, Ja,x = 5.1 Hz, Ja,b = 18.0 Hz, Ha); 3.76 (dd, 1H, Jb,x = 12.0 Hz, Jb,a = 18.0 Hz, Hb); 5.36 (dd, 1H, Jx,a = 5.1 Hz, Jx,b = 12.0 Hz, Hx); 6.51 (sl, 2H, NH); 7.04–7.12 (m, 4H); 7.26 (t, 2H, J = 8,7 Hz); 7.81–7.85 (m, 2H). 13C NMR (75 MHz, DMSO-d6) δ (ppm): 21.0 (CH3); 42.8; 60.1; 116.0 (d, 2 J = 21.7 Hz); 125.8–141.1 (Caromatic); 150.0 (C= ); 155.4 (C=O); 163.3 (d, 1 J = 246.0 Hz).

*5-(4-bromophenyl)-3-(4-fluorophenyl)-4,5-dihydropyrazole-1-carboxamide (****20****).* Yield: 9%; mp: 176.0–178.0 °C; 1H NMR (300 MHz, DMSO-d6) δ (ppm): 3.07 (dd, 1H, Ja,x = 5.7 Hz, Ja,b = 18.6 Hz, Ha); 3.80 (dd, 1H, Jb,x = 12.3 Hz, Jb,a = 18.6 Hz, Hb); 5.40 (dd, 1H, Jx,a = 5.7 Hz, Jx,b = 12.3 Hz, Hx); 6.58 (sl, 2H, NH); 7.15 (m, 2H); 7.28 (t, 2H, J = 9.0 Hz); 7.51 (d, 2H, J = 9.0 Hz); 7.81–7.86 (m, 2H). 13C NMR (75 MHz, DMSO-d6) δ (ppm): 42.1; 59.4; 115.6 (d, 2 J = 21.7 Hz); 119.9–142.9 (Caromatic); 149.7 (C=N); 154.9 (C=O); 163.3 (d, 1 J = 246.0 Hz).

*5-(4-(dimethylamino)phenyl)-3-(4-fluorophenyl)-4,5-dihydropyrazole-1-carboxamide (****21****).* Yield: 17%; mp: 183.1–185.1 °C; 1H NMR (300 MHz, DMSO-d6) δ (ppm): 2.82 (s, 6H, CH3); 3.00 (dd, 1H, Ja,x = 4.8 Hz, Ja,b = 17.7 Hz, Ha); 3.71 (dd, 1H, Jb,x = 11.4 Hz, Jb,a = 17.7 Hz, Hb); 5.29 (dd, 1H, Jx,a = 4.8 Hz, Jx,b = 11.4 Hz, Hx); 6.44 (sl, 2H, NH); 6.63–7.29 (m, 4H); 7.26 (t, 2H, J = 9.0 Hz); 7.80–7.84 (m, 2H).

*5-(4-chlorophenyl)-3-(4-fluorophenyl)-4,5-dihydropyrazole-1-carboxamide (****22****).* Yield: 15%; mp: 149–151 °C; 1H NMR (300 MHz, DMSO-d6) δ (ppm): 3.05 (dd, 1H, Ja,x = 5.4 Hz, Ja,b = 18.0 Hz, Ha); 3.79 (dd, 1H, Jb,x = 12.6 Hz, Jb,a = 18.0 Hz, Hb); 5.40 (dd, 1H, Jx,a = 5.4 Hz, Jx,b = 12.6 Hz, Hx); 6.57 (sl, 2H, NH); 7.18–7.85 (m, 8H, Ar).

*4,5-dihydro-5-(4-(hexyloxy)phenyl)-3-phenylpyrazole-1-carbothioamide (****23****).* Yield: 29%; 1H NMR (300 MHz, DMSO-d6) δ (ppm): 0.84 (sl, 3H, CH3); 1.25–1.67 (m, 8 H, –CH2–); 3.06–3.12 (m, 1H, Ha); 3.79–3.89 (m, 3H, Hb, –OCH2–); 5.83–5.87 (m, 1H, Hx); 6.81–8.01 (m, 11H, Ar e NH). 13C NMR (75 MHz, DMSO-d6) δ (ppm): 13.8–30.9 (Caliphatic); 42.3; 62.3; 67.3 (–OCH2–); 114.2–134.8 (Caromatic); 154.9 (C=N); 157.6; 176.1 (C=S).

*4,5-dihydro-5-(4-(octyloxy)phenyl)-3-phenylpyrazole-1-carbothioamide (****24****).* Yield: 27%; mp: 90.4–92.1 °C; 1H NMR (300 MHz, DMSO-d6) δ (ppm): 0.83 (sl, 3H, CH3); 1.20–1.67 (m, 12 H, –CH2–); 3.07–3.13 (m, 1H, Ha); 3.80–3.90 (m, 3H, Hb, -OCH2-); 5.86 (m, 1H, Hx); 6.81–8.01 (m, 11H, Ar e NH). 13C NMR (75 MHz, DMSO-d6) δ (ppm): 13.8–31.1 (Caliphatic); 42.3; 62.3; 67.3 (–OCH2–); 114.2–134.8 (Caromatic); 154.9 (C=N); 157.6; 176.1 (C=S).

*4,5-dihydro-5-(4-(nonyloxy)phenyl)-3-phenylpyrazole-1-carbothioamide (****25****).* Yield: 14%; mp: 86.4–88.2 °C; 1H NMR (300 MHz, DMSO-d6) δ (ppm): 0.83 (sl, 3H, CH3); 1.22–1.67 (m, 14 H, –CH2–); 3.09 (dd, 1H, Ja,x = 3.0 Hz, Ja,b = 18.0 Hz, Ha); 3.80–3.89 (m, 3H, Hb, –OCH2–); 5.85 (dd, 1H, Jx,a = 3.0 Hz, Jx,b = 11.4 Hz, Hx); 6.81–8.02 (m, 11H, Ar e NH). 13C NMR (75 MHz, DMSO-d6) δ (ppm): 13.9–31.2 (Caliphatic); 42.3; 62.3; 67.3 (–OCH2–); 114.2–134.8 (Caromatic); 154.9 (C=N); 157.6; 176.0 (C=S).

*5-(4-(decyloxy)phenyl)-4,5-dihydro-3-phenylpyrazole-1-carbothioamide (****26****).* Yield: 29%; mp: 96.2–98.9 °C; 1H NMR (300 MHz, DMSO-d6) δ (ppm): 0.83 (t, 3H, J = 6.9 Hz, CH3); 1.22–1.67 (m, 16 H, -CH2-); 3.09 (dd, 1H, Ja,x = 3.0 Hz, Ja,b = 18.0 Hz, Ha); 3.80–3.90 (m, 3H, Hb, -OCH2-); 5.85 (dd, 1H, Jx,a = 3.0 Hz, Jx,b = 11.4 Hz, Hx); 6.81–8.00 (m, 11H, Ar e NH). 13C NMR (75 MHz, DMSO-d6) δ (ppm): 13.8–31.2 (Caliphatic); 42.3; 62.3; 67.3 (–OCH2–); 114.2–134.8 (Caromatic); 154.9 (C= ); 157.6; 176.1 (C=S).

*5-(4-(dodecyloxy)phenyl)-4,5-dihydro-3-phenylpyrazole-1-carbothioamide (****27****).* Yield: 22%; mp: 94.2 °C 96.3 °C; 1H NMR (300 MHz, DMSO-d6) δ (ppm): 0.83 (t, 3H, J = 6.0 Hz, CH3); 1.21–1.69 (m, 20 H, –CH2–); 3.09 (dd, 1H, Ja,x = 3.3 Hz, Ja,b = 18.0 Hz, Ha); 3.80–3.89 (m, 3H, Hb, –OCH2–); 5.86 (dd, 1H, , Jx,a = 3.3 Hz, Jx,b = 11.1 Hz, Hx); 6.81–8.03 (m, 11H, Ar e NH). 13C NMR (75 MHz, DMSO-d6) δ (ppm): 13.9–31.2 (Caliphatic); 42.3; 62.3; 67.3 (–OCH2–); 114.2–134.8 (Caromatic); 154.9 (C=N); 157.6; 176.0 (C=S).

*4,5-dihydro-3-phenyl-5-(4-(tetradecyloxy)phenyl)pyrazole-1-carbothioamide (****28****).* Yield: 37%; mp: 97.0–99.2 °C; 1H NMR (300 MHz, DMSO-d6) δ (ppm): 0.83 (t, 3H, J = 6.9 Hz, CH3); 1.21–1.67 (m, 24 H, –CH2–); 3.12 (dd, 1H, Ja,x = 3.3 Hz, Ja,b = 18.3 Hz, Ha); 3.80–3.90 (m, 3H, Hb, –OCH2–); 5.85 (dd, 1H, Jx,a = 3.3 Hz, Jx,b = 11.1 Hz); 6.81–8.00 (m, 11H, Ar and NH). 13CNMR (75 MHz, DMSO-d6) δ (ppm): 13.8–31.2 (Caliphatic); 42.3; 62.3; 67.3 (-OCH2-); 114.2–134.8 (Caromatic); 154.9 (C=N); 157.6; 176.1 (C=S).

*3-(4-fluorophenyl)-4,5-dihydro-5-(4-(nonyloxy)phenyl)pyrazole-1-carbothioamide (****29****).* Yield: 25%; mp: 113–114 °C; 1H NMR (300 MHz, DMSO-d6) δ (ppm): 0.81 (t, 3H, J = 6.0 Hz, CH3); 1.21–1.65 (m, 14 H, –CH2–); 3.05–3.11 (m, 1H, Ha); 3.78–3.87 (m, 3H, Hb, –OCH2-); 5.84–5.87 (m, 1H, Hx); 6.80 (d, 4H, J = 9.0 Hz); 7.02 (m, 2H); 7.27 (t, 2H, J = 9.0 Hz); 7.89–8.02 (m, 4H, Ar e NH). 13C NMR (75 MHz, DMSO-d6) δ (ppm): 13.9–31.3 (Caliphatic); 42.5; 62.4; 67.3 (–OCH2–); 114.2; 115.8 (d, 2 J = 21.7 Hz); 126.6–134.8 (Caromatic); 154.0 (C=N); 157.6; 163.3 (d, 1 J = 246.7 Hz); 176.1 (C=S).

*3-(4-fluorophenyl)-4,5-dihydro-5-(4-(decyloxy)phenyl)pyrazole-1-carbothioamide (****30****).* Yield: 23%; mp: 105.5–108.4 °C; 1H NMR (300 MHz, DMSO-d6) δ (ppm): 0.82 (t, 3H, J = 6.3 Hz, CH3); 1.21–1.67 (m, 24 H, –CH2–); 3.09 (dd, 1H, Ha, Ja,x = 3.0 Hz, Ja,b = 18.3 Hz); 3.79–3.89 (m, 3H, Hb, –OCH2–); 5.85 (dd, 1H, Jx,a = 3.0 Hz, Jx,b = 9.9 Hz, Hx); 6.80 (d, 4H, J = 8.7 Hz); 7.03 (m, 2H, J = 8.7 Hz); 7.28 (t, 2H, J = 8.7 Hz); 7.90–8.01 (m, 4H, Ar and NH). 13C NMR (75 MHz, DMSO-d6) δ (ppm): 13.9–31.2 (Caliphatic); 42.4; 62.4; 67.3 (–OCH2–); 114.2; 115.7 (d, 2 J = 21.5 Hz); 126.6–134.8 (Caromatic); 154.0 (C=N); 157.6; 163.4 (d, 1 J = 247.3 Hz); 176.1 (C=S).

*3-(4-fluorophenyl)-4,5-dihydro-5-(4-(tetradecyloxy)phenyl)pyrazole-1-carbothioamide (****31****)*. Yield: 26%; mp: 92.2–94.6 °C; 1H NMR (300 MHz, DMSO-d6) δ (ppm): 0.78 (sl, 3H, CH3); 1.16–1.60 (m, 24 H, –CH2–); 3.00–3.06 (m, 1H, Ha); 3.81 (sl, 3H, Hb, –OCH2–); 5,71–5.86 (m, 1H, Hx); 6.75–8.00 (m, 10H, Ar and NH).

### Preparation of pyrazoles derivatives (32–34)

The pyrazoles were synthesized according to a previously published procedure^36^. A solution of 0.83 mmol (1 eq.) of the respective chalcone in 10 mL of ethanol, 1.00 mmol (1.2 eq.) of TsNHNH2 and 2 mol% iodine was added in a round bottom flask. The mixture was left under magnetic stirring and reflux for 10 min. After that time, 1.25 mmol (1.5 eq.) of K_2_CO_3_ was added to the mixture. The reaction was monitored by TLC (eluent: 100% DCM, revelator: ultraviolet light and I2 vapor). After completion, the mixture was extracted with ethyl acetate and 5% Na_2_S_2_O_3_ solution. The organic layer was dried with Na_2_SO_4_ and then filtered and concentrated under reduced pressure. DCM or DCM/hexane were added to the crude and the products were vacuum filtered affording the desired compounds as pure solids. The 1H NMR spectra of compounds **32**–**34** are shown in Supplementary Information.

*5-(4-fluorophenyl)-3-(4-methylphenyl)-1H-pyrazole (****32****).* Yield: 16%; m.p.: decomposes at 302 °C; 1H NMR (DMSO-d6; 500 MHz) δ ppm: 13.30 (1H, brs, NH), 7.88 (2H, s), 7.70 (2H, s), 7.26 (4H, s), 7.10 (1H, s, Hx). 13C NMR (DMSO-d6; 125 MHz) δ ppm: 162.7, 160.8, 150.4, 143.6, 137.6, 130.3, 129.5, 127.1, 126.5, 125.1, 115.6, 115.4, 99.2 (C-Hx), 20.8 (CH3).

*3-(4-bromophenyl)-5-(4-fluorophenyl)-1H-pyrazole (****33****).*Yield: 30%; m.p.: 217–219 °C; 1H NMR (DMSO-d6; 500 MHz) δ ppm: 13.44 (1H, brs, NH), 7.85–7.79 (4H, m), 7.71–7.63 (2H, m), 7.31–7.28 (2H, m), 7.20 (1H, s, Hx). 13C NMR (DMSO-d6; 125 MHz) δ ppm: 162.8, 160.9, 150.3, 142.7, 132.8, 131.6, 130.0, 128.4, 127.1, 125.8, 120.5, 115.9, 115.6, 99.9 (C-Hx).

*3-(4-chlorophenyl)-5-(4-fluorophenyl)-1H-pyrazole (****34****).* Yield: 6%; m.p.: decomposes at 193 °C; 1H NMR (DMSO-d6; 500 MHz) δ ppm: 13.43 (1H, brs, NH), 7.87–7.83 (4H, m), 7.54–7.48 (2H, m), 7.33–7.26 (2H, m), 7.20 (1H, s, Hx). 13C NMR (DMSO-d6; 125 MHz) δ ppm: 162.9, 160.9, 150.6, 150.2, 142.7, 132.5, 132.0, 129.1, 128.7, 127.3, 126.8, 116.1, 116.0, 115.6, 115.5, 99.9 (C-Hx).

### Drugs, media, and reagents for biological assays

Dulbecco’s Modified Eagle Medium (DMEM), Roswell Park Memorial Institute (RPMI) 1640 medium, inactivated fetal bovine serum, and antibiotics (10,000 U/mL penicillin G sodium salt, and 10 mg/mL streptomycin sulfate) were purchased from Atena Biotecnologia (Vitrocell, Campinas, SP, Brazil). Thiazolyl blue tetrazolium bromide (MTT) and dimethyl sulfoxide (DMSO) were obtained from Sigma-Aldrich (St. Louis, MO, USA). Praziquantel was kindly provided by Ecovet Indústria Veterinária Ltda (São Paulo, SP, Brazil). In all procedure, compounds were solubilized in DMSO.

### Preparation of parasites and in vitro antischistosomal assay

Adult schistosomes (BH strain) were isolated from infected mice and cultured in RPMI medium + 10% fetal calf serum and 100 U/mL penicillin and 100 μg/mL streptomycin in a 24-well culture plate (Corning, New York, NY, USA) containing one pair of parasites per well at 37 °C and 5% CO_2_ (Panasonic Healthcare, Sakata, Oizumi-machi, Japan)^38,39^. For antischistosomal assay, compounds were dissolved in DMSO (final concentration of 0.5% v/v). Each drug concentration was tested at least in triplicate, and the experiments were repeated three times. The initial concentration of the pyrazolines and praziquantel was 50 μM and 3 μM, respectively, which was serially diluted in medium with twofold dilutions to give six concentrations^40,41^. The negative control worms were assayed in RPMI 1640 medium and RPMI 1640 with 0.5% DMSO. Parasites were monitored daily at 24, 48 and 72 h under a stereomicroscope (Leica Microsystems EZ4E, Wetzlar, Germany) and an inverted microscope (BEL Engineering INV 100, Monza, MB, Italy). Egg production was evaluated daily as previously described^42^. Parasite viability was averaged and 50% effective concentrations (EC_50_) were calculated using GraphPad Prism software^24,43^.

### Cell culture and cytotoxicity assay

The cytotoxicity of compounds was tested against Vero cells (African green monkey kidney cells obtained from the American Type Culture Collection, ATCC; Manassas, VA, USA) and SH-SY5Y cells (human neuroblastoma obtained from Banco de Células do Rio de Janeiro, BCRJ, RJ, Brazil). Cells was cultured in DMEM medium supplemented with 10% fetal calf serum, penicillin (100 U/mL) and streptomycin (100 µg/mL) and 2 mM of l-glutamine at 37 °C and in a 5% CO_2_ humidified incubator (Panasonic). The DMEM contained 1 or 4 mg/mL glucose for Vero and SH-SY5Y cells, respectively. Cytotoxicity assay was performed as previously described^25,26^. Briefly, cells were seeded in 96-well plates (Corning) in DMEM medium and incubated with twofold serial drug starting at 200 μM following a 24 h adhesion period at 37 °C in 5% CO_2_. After 72 h, MTT was added and incubation was continued for another 3 h. The plate was then read using spectrophotometer (Epoch Microplate Spectrophotometer, BioTek Instruments, Winooski, VT, USA) at 595 nm. The assay was conducted in duplicate and repeated three times. Values were expressed as percentage of the control and 50% cytotoxic concentration (CC_50_) values were calculated^27^. The selectivity indices (SI) of tested compounds were calculated by dividing CC_50_ values obtained on mammalian cells with EC_50_ values determined on *S. mansoni*^44^.

### In silico studies and drug-likeness assessment

Physicochemical descriptors as well as prediction of pharmacokinetic parameters, druglike nature and medicinal chemistry friendliness of compounds **16, 18, 19, 21, 22** and **23** were obtained using the webserver SwissADME^45^. Drug-likeness with filters including Lipinski (Pfizer), Veber (GSK), Muegge (Bayer), Egan (Pharmacia), and Ghose (Amgen) were performed using the same webserver.

### Ethical approval

The study was conducted according to the guidelines of Animal Ethics and approved by the Committee for the Ethical Use of Animals in Experimentation of Guarulhos University (São Paulo, Brazil) according to Brazilian law (protocol code 31/17, approved on 20 March 2017).

## Supplementary Information

Supplementary Information.

## References

[1] McManus DP, Dunne DW, Sacko M, Utzinger J, Vennervald BJ, Zhou XN (2018). Schistosomiasis. Nat. Rev. Dis. Primers.

[2] Colley DG, Bustinduy AL, Secor WE, King CH (2014). Human schistosomiasis. Lancet.

[3] World Health Organization. *Schistosomiasis* (2021). https://www.who.int/news-room/fact-sheets/detail/schistosomiasis.

[4] Mawa PA, Kincaid-Smith J, Tukahebwa EM, Webster JP, Wilson S (2021). Schistosomiasis morbidity hotspots: Roles of the human host, the parasite and their interface in the development of severe morbidity. Front. Immunol..

[5] Wiegand RE (2017). A persistent hotspot of *Schistosoma mansoni* infection in a five-year randomized trial of praziquantel preventative chemotherapy strategies. J. Infect. Dis..

[6] Deol AK (2019). Schistosomiasis—Assessing progress toward the 2020 and 2025 global goals. N. Engl. J. Med..

[7] Assaré RK (2020). Characteristics of persistent hotspots of *Schistosoma mansoni* in western Côte d'Ivoire. Parasit Vectors.

[8] Kabuyaya M, Chimbari MJ, Mukaratirwa S (2018). Efficacy of praziquantel treatment regimens in pre-school and school aged children infected with schistosomiasis in sub-Saharan Africa: A systematic review. Infect. Dis. Poverty.

[9] Chelladurai JJ, Kifleyohannes T, Scott J, Brewer MT (2018). Praziquantel resistance in the zoonotic cestode *Dipylidium caninum*. Am. J. Trop. Med. Hyg..

[10] Mafud AC, Ferreira LG, Mascarenhas YP, Andricopulo AD, de Moraes J (2016). Discovery of novel antischistosomal agents by molecular modeling approaches. Trends Parasitol..

[11] Lago EM, Xavier RP, Teixeira TR, Silva LM, da Silva Filho AA, de Moraes J (2018). Antischistosomal agents: State of art and perspectives. Future Med. Chem..

[12] Vitaku E, Smith DT, Njardarson JT (2014). Analysis of the structural diversity, substitution patterns, and frequency of nitrogen heterocycles among U.S. FDA approved pharmaceuticals. J. Med. Chem..

[13] de Moraes J, Geary TG (2020). FDA-Approved antiparasitic drugs in the 21st century: A success for helminthiasis?. Trends Parasitol..

[14] Rando DGG (2019). Vanillin-related N-acylhydrazones: Synthesis, antischistosomal properties and target fishing studies. Curr. Top. Med. Chem..

[15] Amorim CR (2020). Schiff bases of 4-Phenyl-2-Aminothiazoles as hits to new antischistosomal: Synthesis, *in vitro, in vitro* and *in silico* studies. Eur. J. Pharm. Sci..

[16] Havrylyuk D, Roman O, Lesyk R (2016). Synthetic approaches, structure activity relationship and biological applications for pharmacologically attractive pyrazole/pyrazoline–thiazolidine-based hybrids. Eur. J. Med. Chem..

[17] Kumar G (2018). Pyrazole–pyrazoline as promising novel antimalarial agents: A mechanistic study. Eur. J. Med. Chem..

[18] Matiadis D (2021). Pyrazol(in)e derivatives of curcumin analogs as a new class of anti-*Trypanosoma cruzi* agents. Future Med. Chem..

[19] de Moraes J (2015). Natural products with antischistosomal activity. Future Med. Chem..

[20] Pink R, Hudson A, Mouriès MA, Bendig M (2005). Opportunities and challenges in antiparasitic drug discovery. Nat. Ver. Drug Discov..

[21] Patra M (2012). Ferrocenyl derivatives of the anthelmintic praziquantel: Design, synthesis, and biological evaluation. J. Med. Chem..

[22] Mayoka G, Keiser J, Häberli C, Chibale K (2019). Structure–activity relationship and *in vitro* absorption, distribution, metabolism, excretion, and toxicity (ADMET) studies of N-aryl 3-trifluoromethyl pyrido[1,2- a]benzimidazoles that are efficacious in a mouse model of schistosomiasis. ACS Infect. Dis..

[23] Menezes CM (2012). Synthesis, biological evaluation, and structure–activity relationship of clonazepam, meclonazepam, and 1,4-benzodiazepine compounds with schistosomicidal activity. Chem. Biol. Drug Des..

[24] Roquini DB (2019). Promethazine exhibits antiparasitic properties *in vitro* and reduces worm burden, egg production, hepato-, and splenomegaly in a schistosomiasis animal model. Antimicrob. Agents Chemother..

[25] Sessa DP, Mengarda AC, Simplicio PE, Antar GM, Lago JHG, de Moraes J (2020). 15β-Senecioyl-oxy-ent-kaur-16-en-19-oic acid, a diterpene isolated from Baccharis lateralis, as promising oral compound for the treatment of schistosomiasis. J. Nat. Prod..

[26] Dematei A (2021). Mechanistic insights into the leishmanicidal and bactericidal activities of batroxicidin, a cathelicidin-related peptide from a south american viper (Bothrops atrox). J. Nat. Prod..

[27] de Brito MG (2020). Therapeutic effect of diminazene aceturate on parasitic blood fluke *Schistosoma mansoni* infection. Antimicrob. Agents Chemother..

[28] Baell JB, Holloway GA (2010). New substructure filters for removal of pan assay interference compounds (PAINS) from screening libraries and for their exclusion in bioassays. J. Med. Chem..

[29] Brenk R (2008). Lessons learnt from assembling screening libraries for drug discovery for neglected diseases. ChemMedChem.

[30] Struble TJ (2020). Current and future roles of artificial intelligence in medicinal chemistry synthesis. J. Med. Chem..

[31] Lipinski CA, Lombardo F, Dominy BW, Feeney PJ (2001). Experimental and computational approaches to estimate solubility and permeability in drug discovery and development settings. Adv. Drug Deliv. Rev..

[32] Veber DF, Johnson SR, Cheng HY, Smith BR, Ward KW, Kopple KD (2002). Molecular properties that influence the oral bioavailability of drug candidates. J. Med. Chem..

[33] Egan WJ, Merz KM, Baldwin JJ (2000). Prediction of drug absorption using multivariate statistics. J. Med. Chem..

[34] Muegge I, Heald SL, Brittelli D (2001). Simple selection criteria for drug-like chemical matter. J. Med. Chem..

[35] Ghose AK, Viswanadhan VN, Wendoloski JJ (1999). A knowledge-based approach in designing combinatorial or medicinal chemistry libraries for drug discovery. 1. A qualitative and quantitative characterization of known drug databases. J. Comb. Chem..

[36] Enes KB (2020). Synthesis and theoretical study of a series of 3,5-disubstitutes pyrazoles. Lett. Org. Chem..

[37] Miguel FB, Dantas JA, Amorim S, Andrade GFS, Costa LAS, Couri MRC (2016). Synthesis, spectroscopic and computational characterization of the tautomerism of pyrazoline derivatives from chalcones. Spectrochim. Acta A Mol. Biomol. Spectrosc..

[38] Lago EM (2019). Phenotypic screening of nonsteroidal anti-inflammatory drugs identified mefenamic acid as a drug for the treatment of schistosomiasis. EBioMedicine.

[39] Porto R (2021). Antiparasitic properties of cardiovascular agents against human intravascular parasite *Schistosoma mansoni*. Pharmaceuticals (Basel).

[40] Silva TC (2021). New evidence for tamoxifen as an antischistosomal agent: *In vitro*, *in vitro* and target fishing studies. Future Med. Chem..

[41] Xavier RP (2020). H1-antihistamines as antischistosomal drugs: *In vitro* and *in vitro* studies. Parasit Vectors.

[42] Silva MP (2021). Brazilian red propolis exhibits antiparasitic properties *in vitro* and reduces worm burden and egg production in an mouse model harboring either early or chronic *Schistosoma mansoni* infection. J. Ethnopharmacol..

[43] Guerra RA (2019). *In vitro* and *in vitro* studies of spironolactone as an a antischistosomal drug capable of clinical repurposing. Antimicrob. Agents Chemother..

[44] Mengarda AC (2021). Licarin A, a neolignan isolated from *Nectandra**oppositifolia* Nees & Mart. (Lauraceae), exhibited moderate preclinical efficacy against *Schistosoma**mansoni* infection. Phytother. Res..

[45] Daina A, Michielin O, Zoete V (2017). Swiss ADME: A free web tool to evaluate pharmacokinetics, drug-likeness and medicinal chemistry friendliness of small molecules. Sci. Rep..

