# Supplementary material for: Pyrazoline derivatives as promising novel antischistosomal agents
Source: Sci Rep. 2021 Dec 6;11:23437. doi: 10.1038/s41598-021-02792-0 (PMC8648852; doi:10.1038/s41598-021-02792-0)
Supplement: Supplementary file 1 — Supplementary Information. [file 41598_2021_2792_MOESM1_ESM.pdf]

Pyrazoline derivatives as promising novel antischistosomal agents

Cristiane S. Morais[1], Ana C. Mengarda[1], Fábio B. Miguel[2], Karine B. Enes[2], Vinícius C. Rodrigues[1], Abolghasem Siyadatpanah[3], Polrat Wilairatana[4,*], Mara R. C. Couri[2,*], and Josué de Moraes[1,*]

[1]Research Center for Neglected Diseases, Guarulhos University, Praça Tereza Cristina, 229, Centro, Guarulhos, SP 07023-070, Brazil.
[2]Department of Chemistry, Federal University of Juiz de Fora, Juiz de Fora, MG 36036-900, Brazil.
[3]Ferdows School of Paramedical and Health, Birjand University of Medical Sciences, Birjand, 9717853577, Iran.
[4]Department of Clinical Tropical Medicine, Faculty of Tropical Medicine, Mahidol University, Bangkok 10400, Thailand.

[*]Corresponding authors: moraesnpdn@gmail.com (J.d.M.); polrat.wil@mahidol.ac.th (P.W.); mara.rubia@ufjf.edu.br (M.R.C.C.).

Supplementary Information

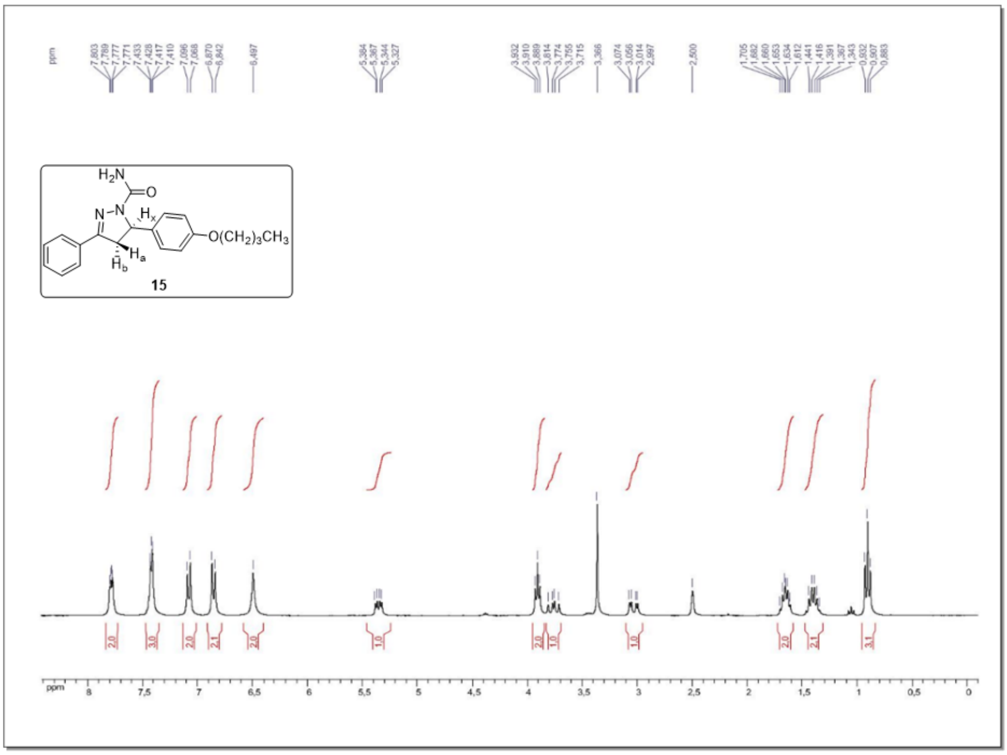

Figure S1. ^{1}H NMR of compound **15**.

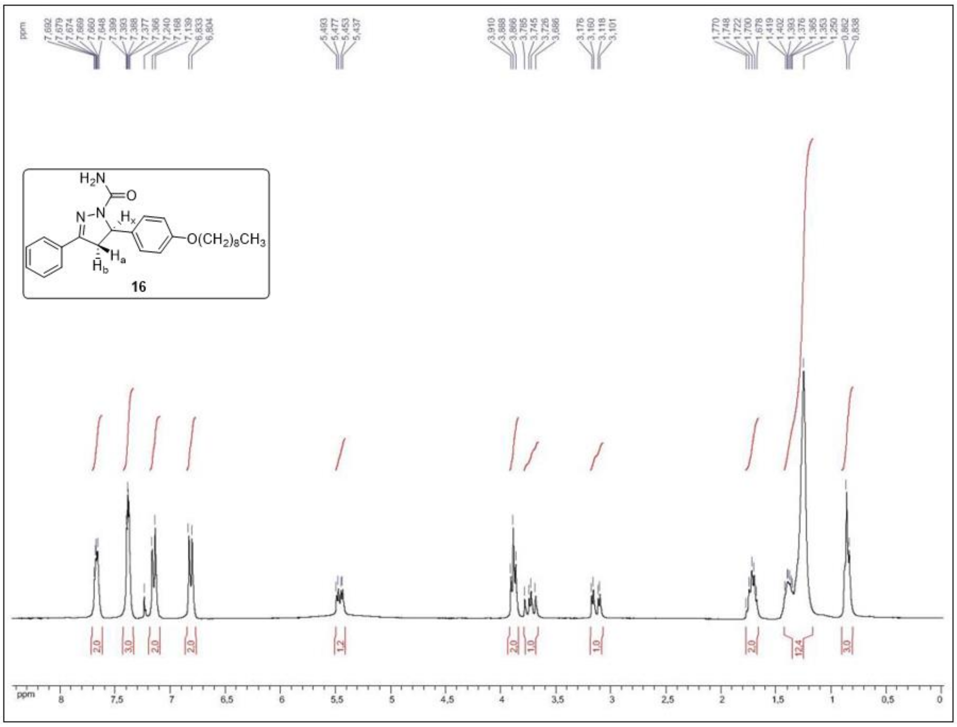

Figure S2. ^{1}H NMR of compound **16**.

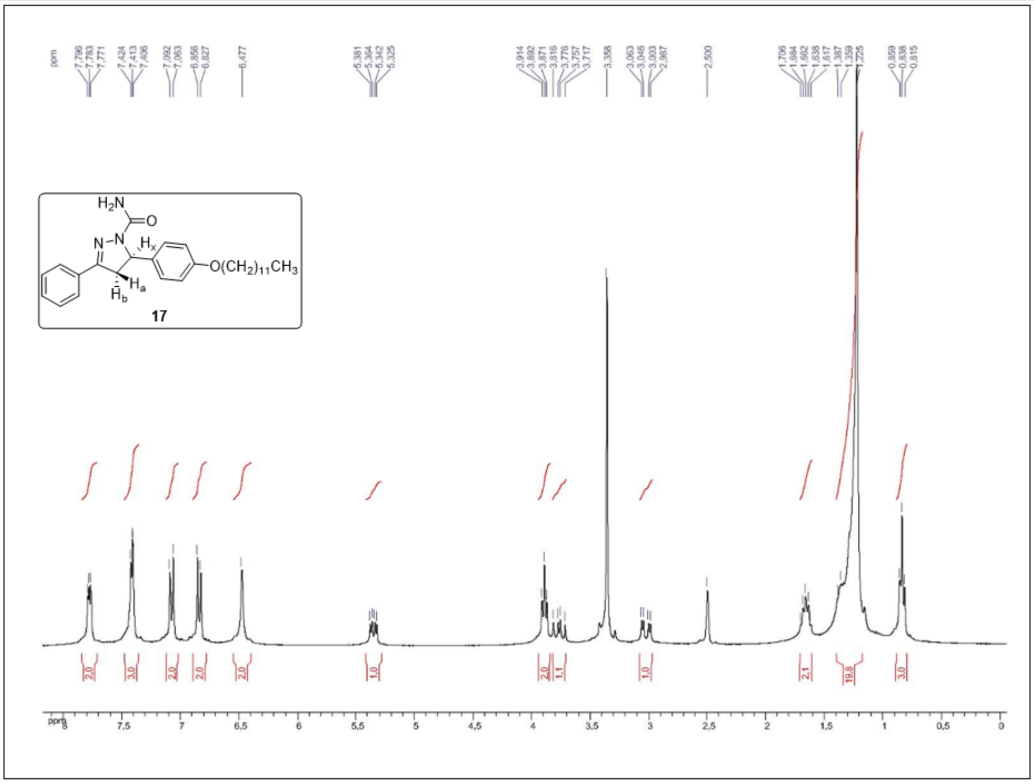

Figure S3. ^{1}H NMR of compound **17**.

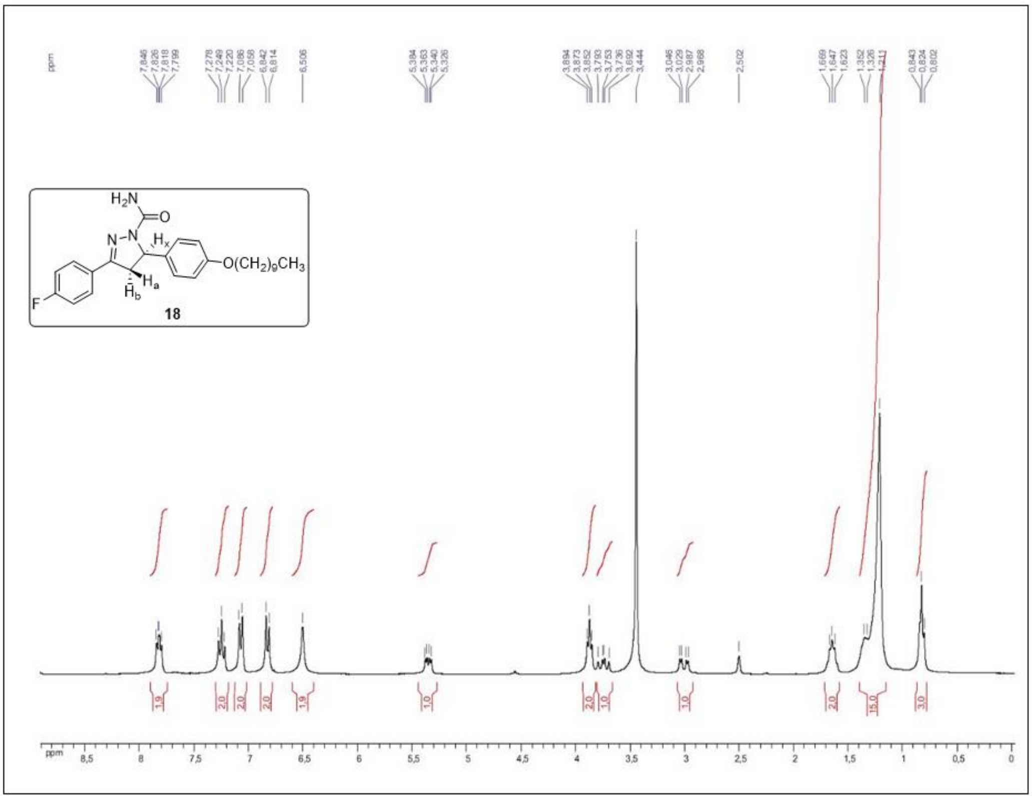

Figure S4. ^{1}H NMR of compound **18**.

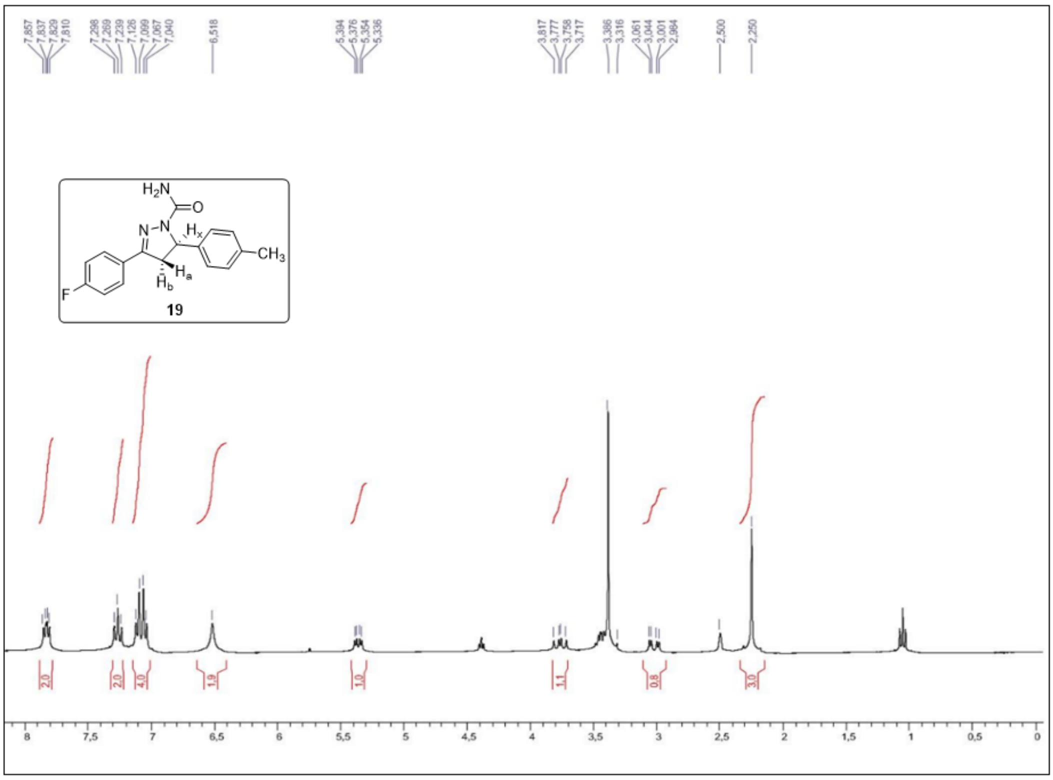

Figure S5. ^{1}H NMR of compound **19**.

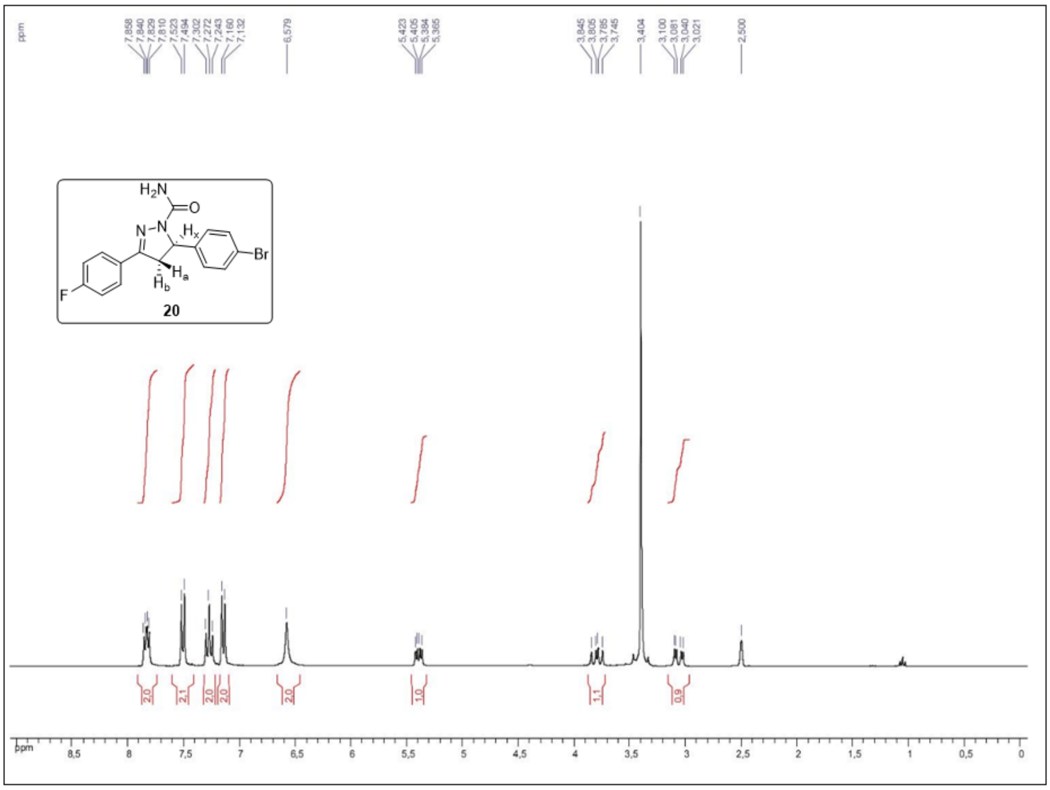

Figure S6. ^{1}H NMR of compound **20**.

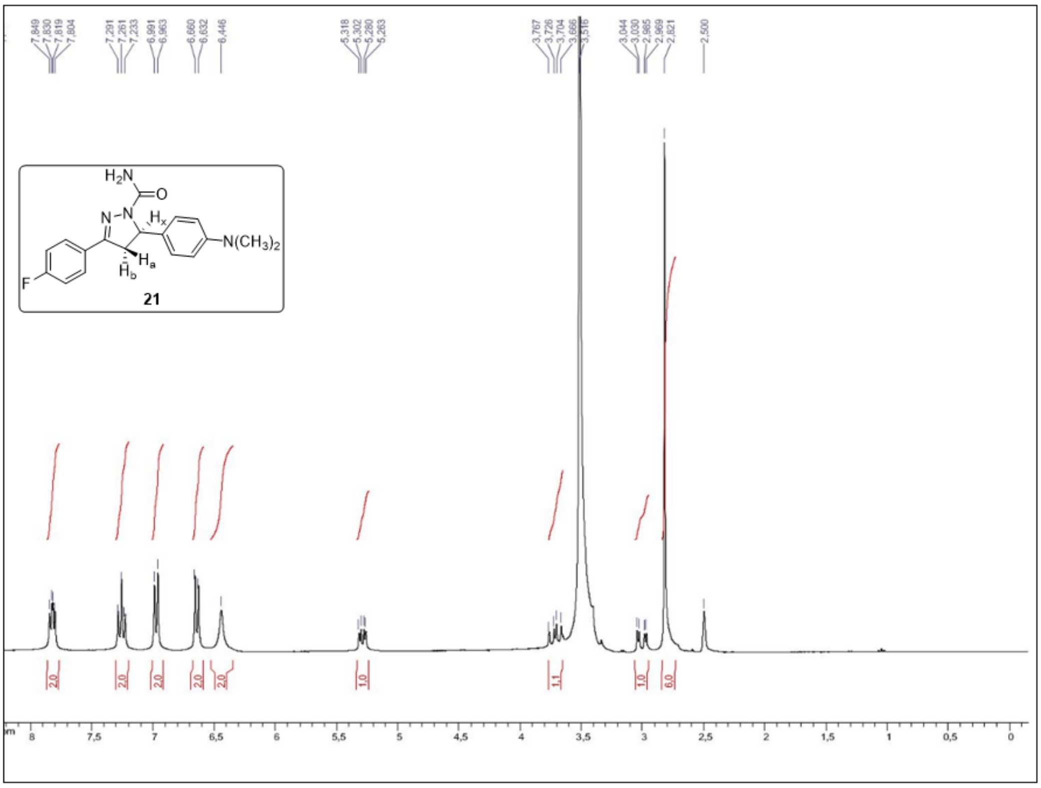

Figure S7. ^{1}H NMR of compound **21**.

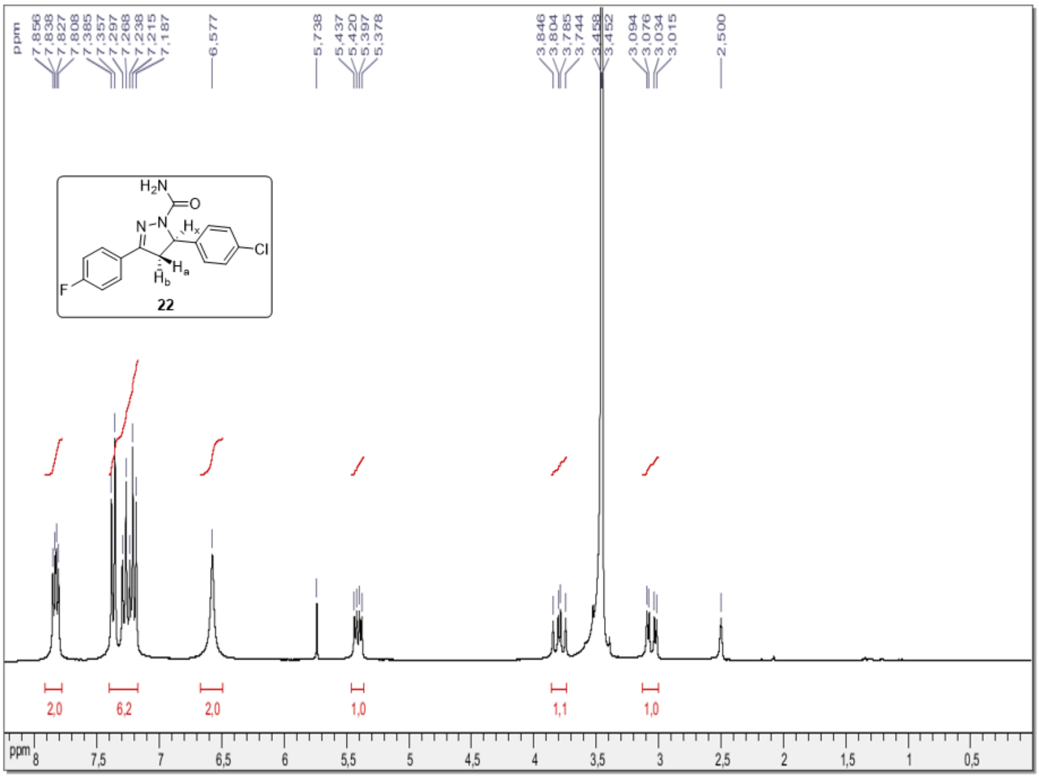

Figure S8. ^{1}H NMR of compound **22**.

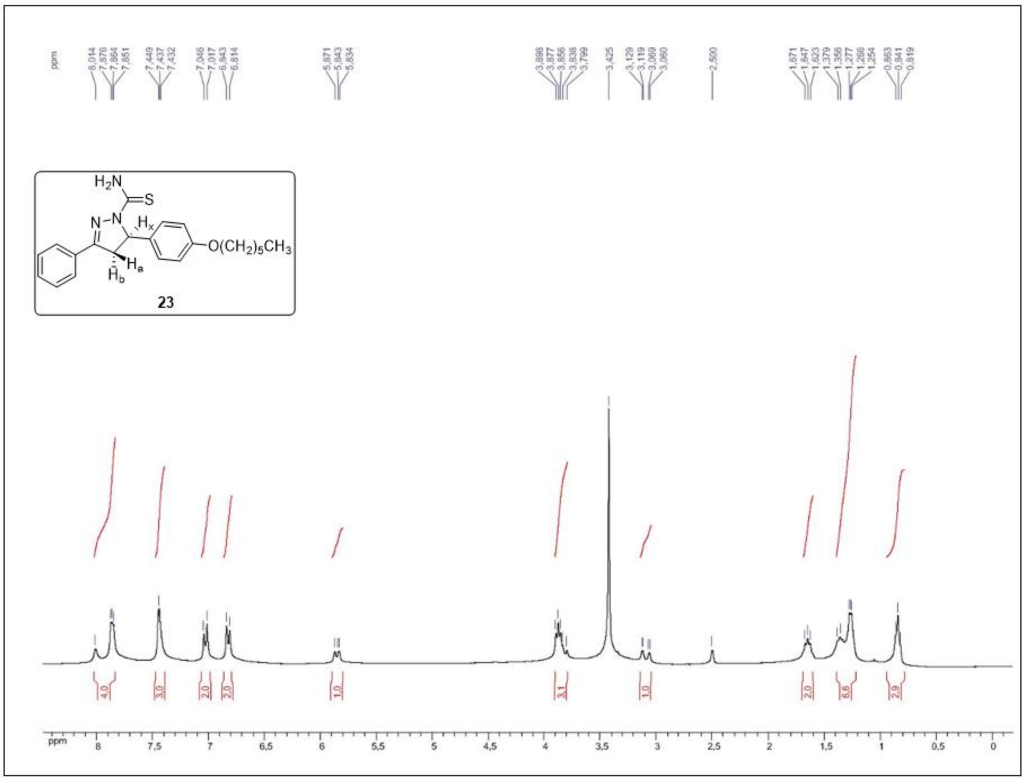

Figure S9. ^{1}H NMR of compound **23**.

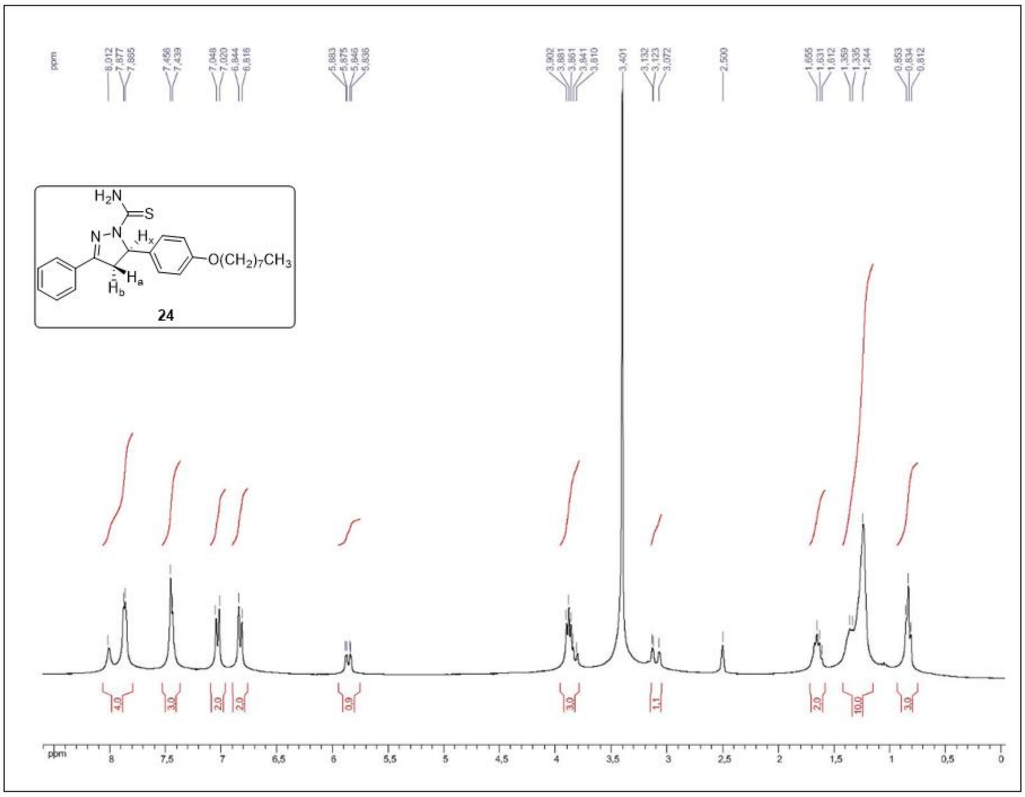

Figure S10. ^{1}H NMR of compound **24**.

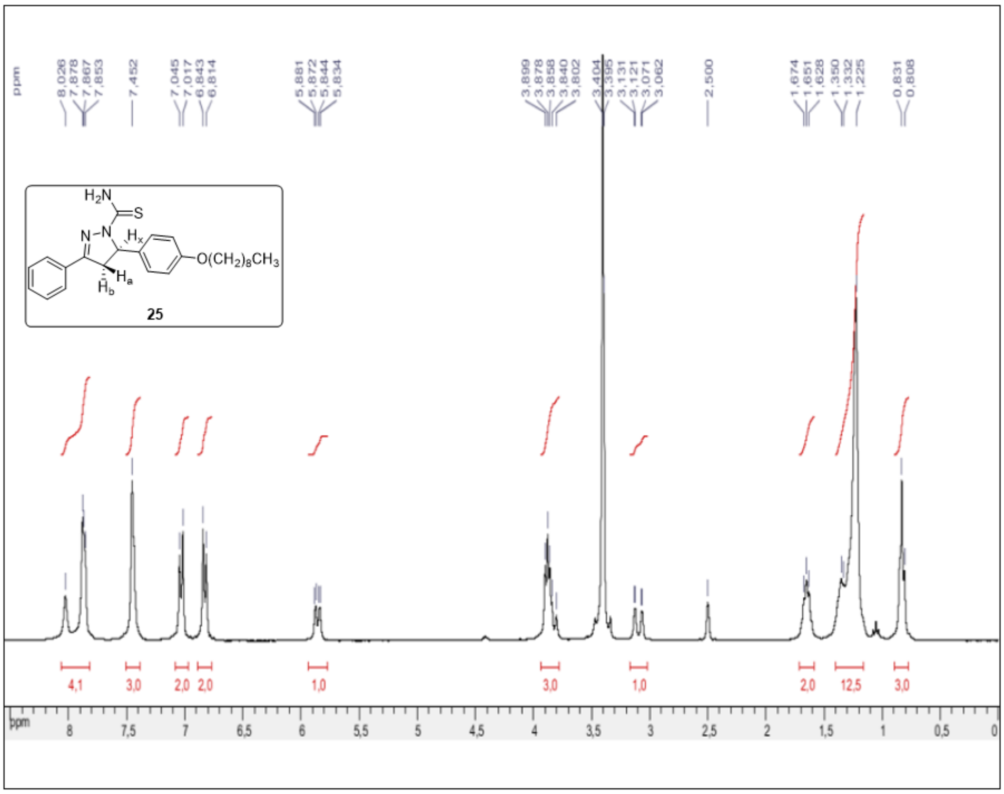

Figure S11. ^{1}H NMR of compound **25**.

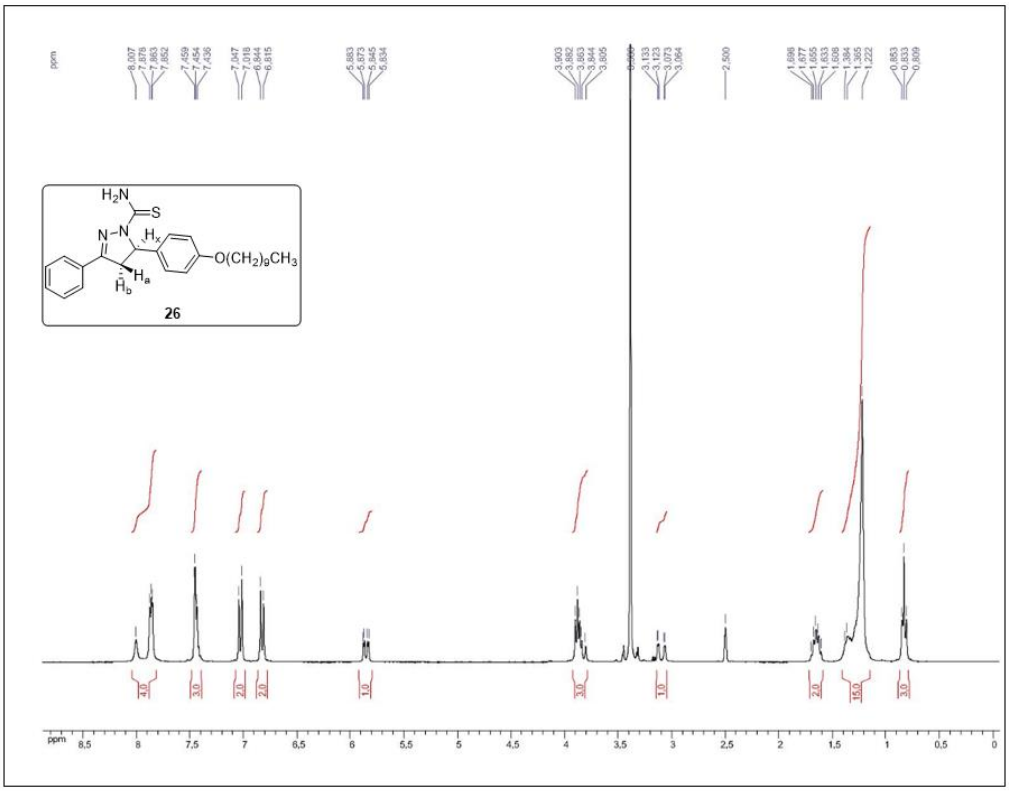

Figure S12. ^{1}H NMR of compound **26**.

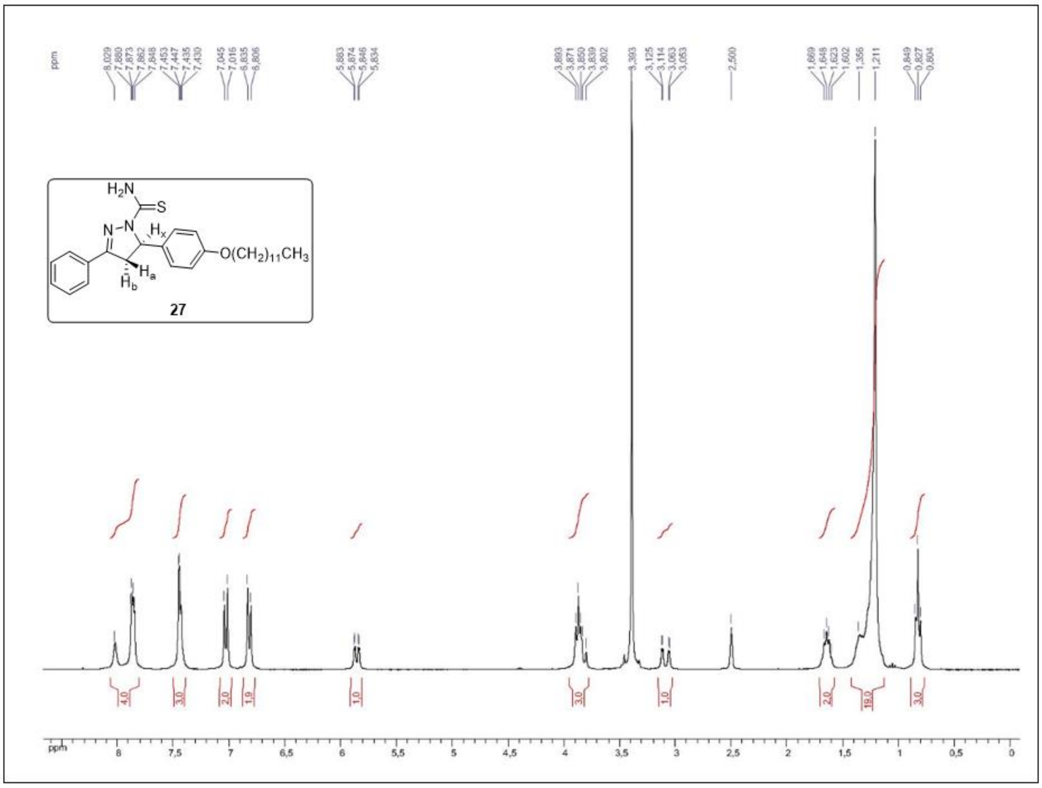

Figure S13. ^{1}H NMR of compound **27**.

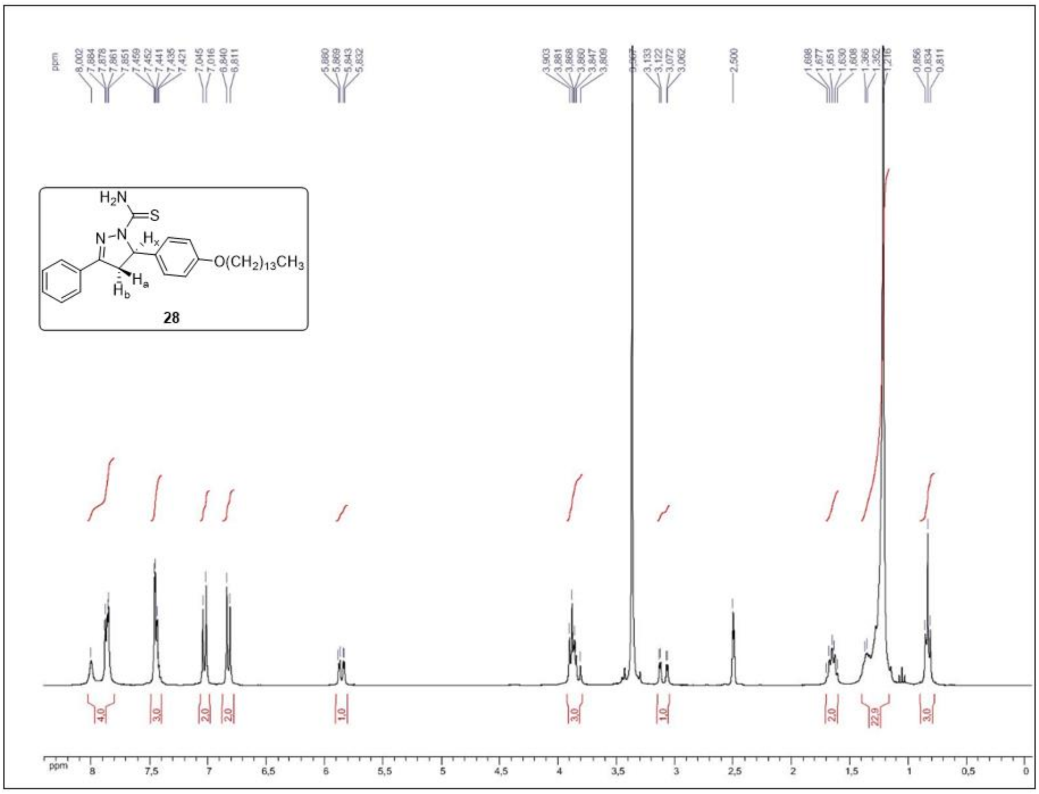

Figure S14. ^{1}H NMR of compound **28**.

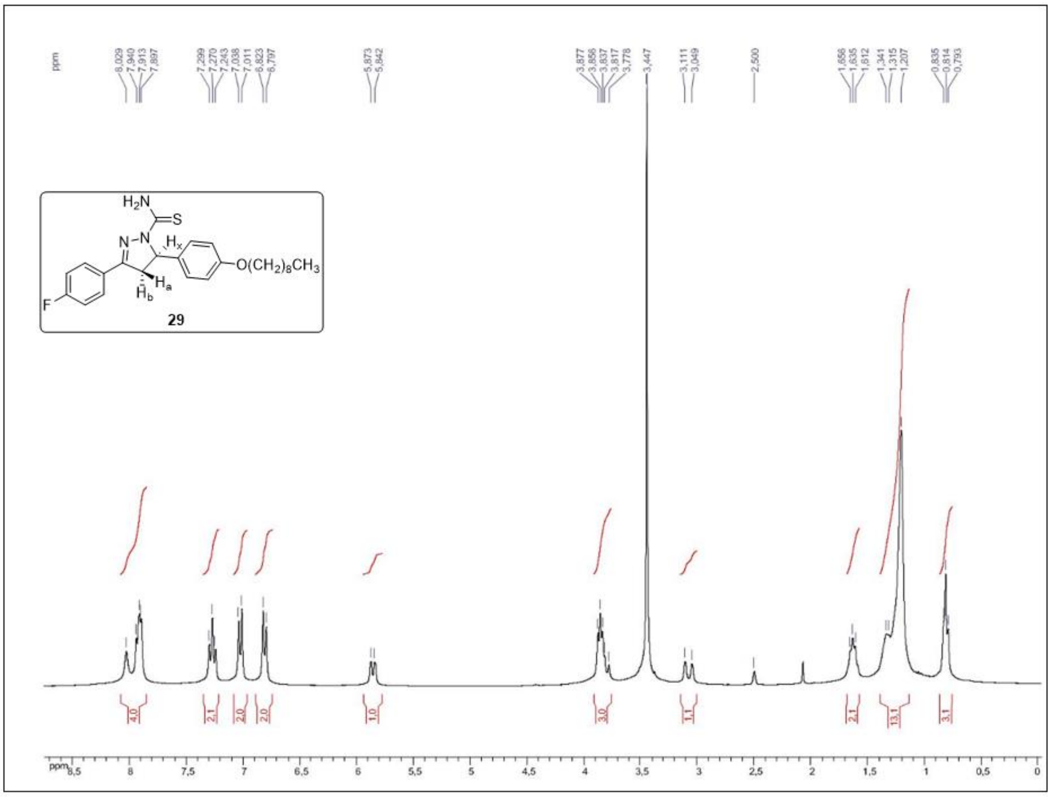

Figure S15. ^{1}H NMR of compound **29**.

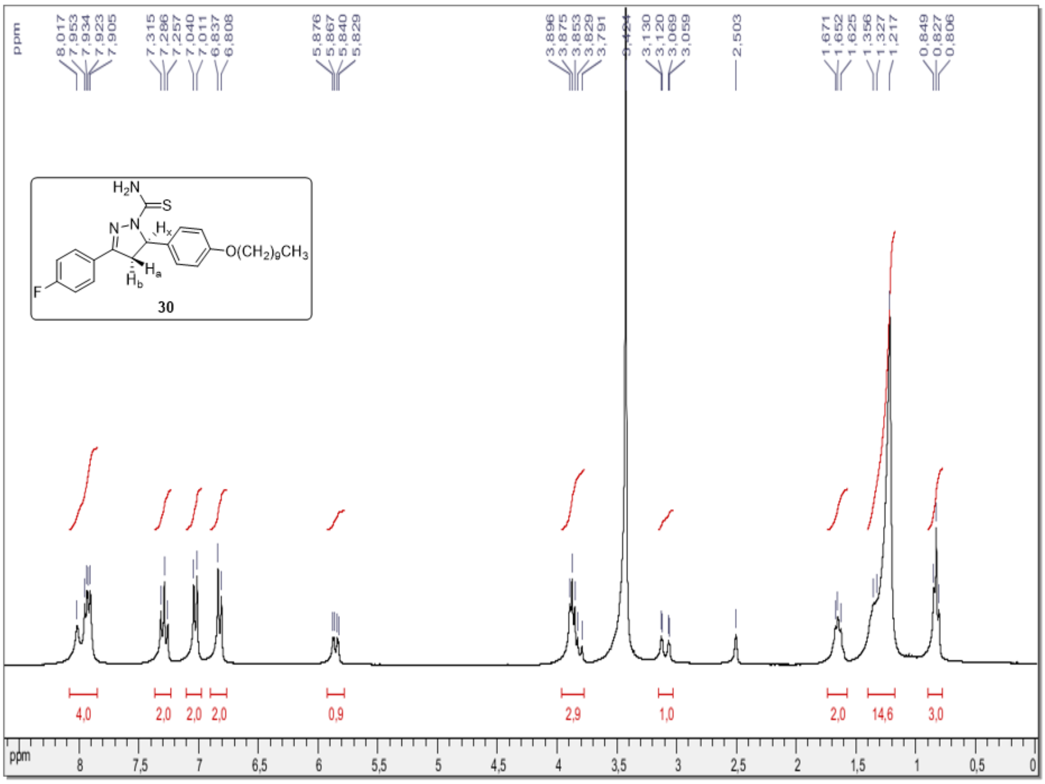

Figure S16. ^{1}H NMR of compound **30**.

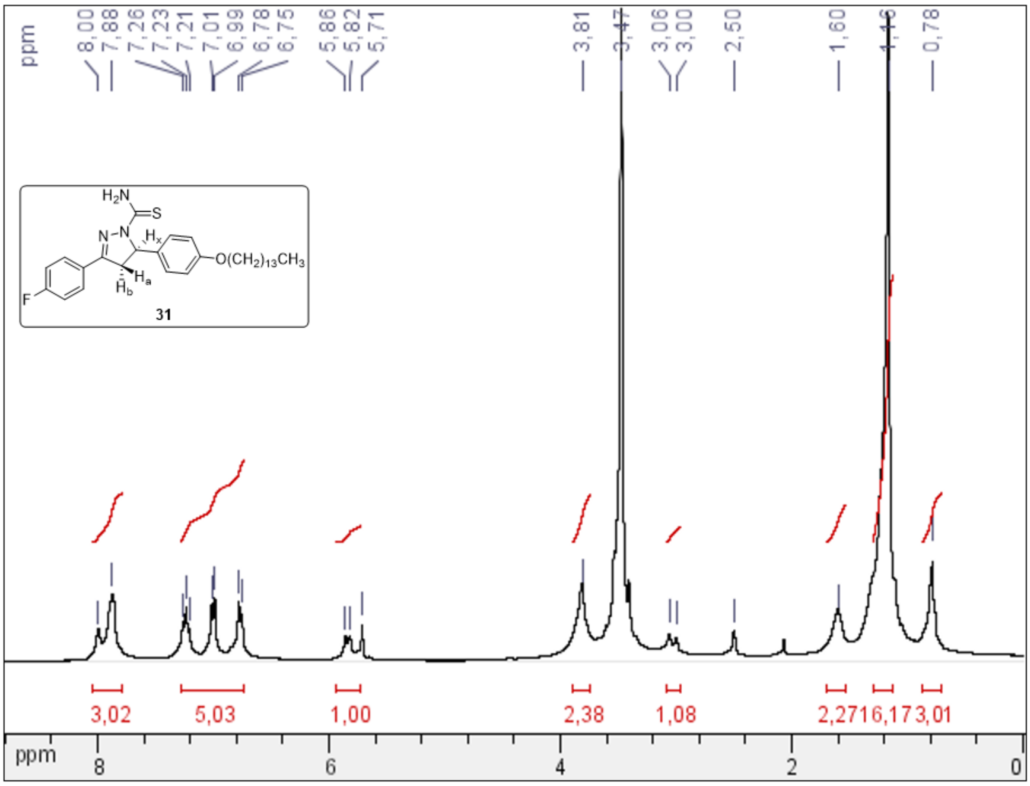

Figure S17. ^{1}H NMR of compound **31**.

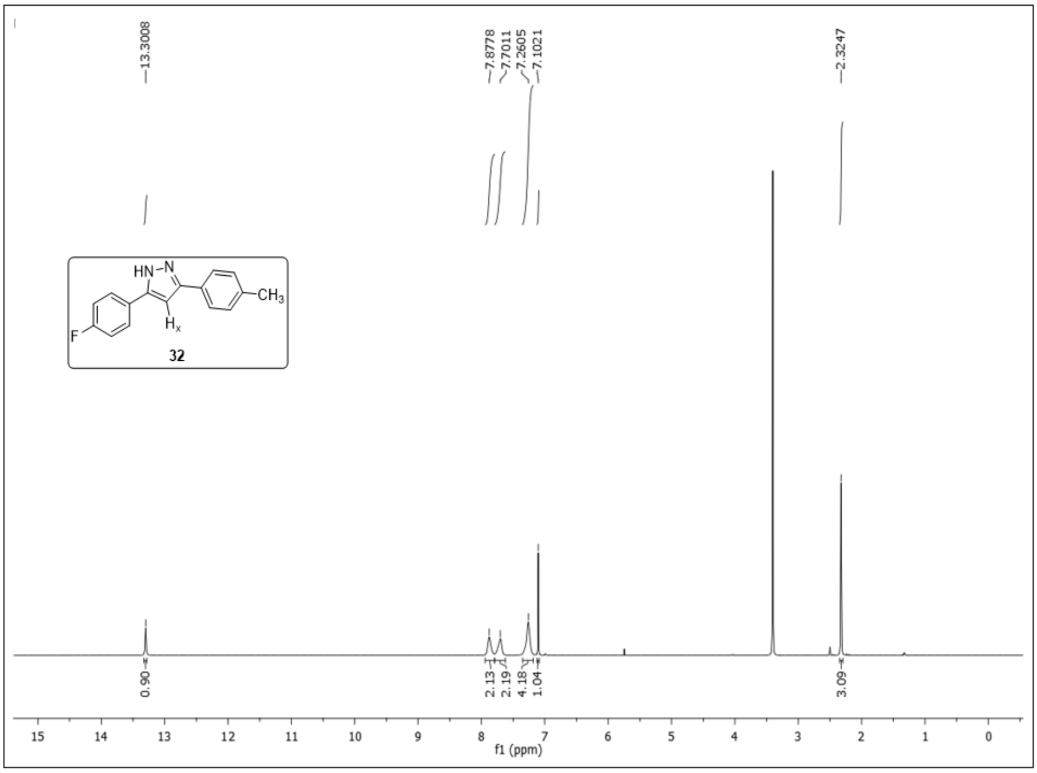

Figure S18. ^{1}H NMR of compound **32**.

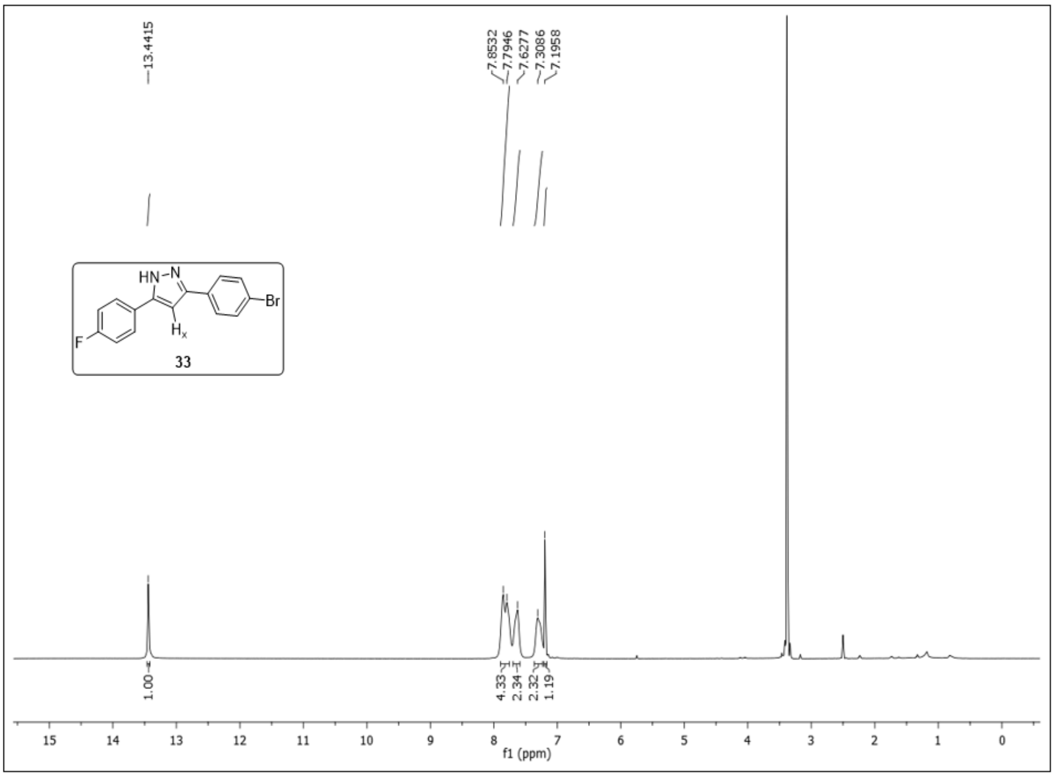

Figure S19. ^{1}H NMR of compound **33**.

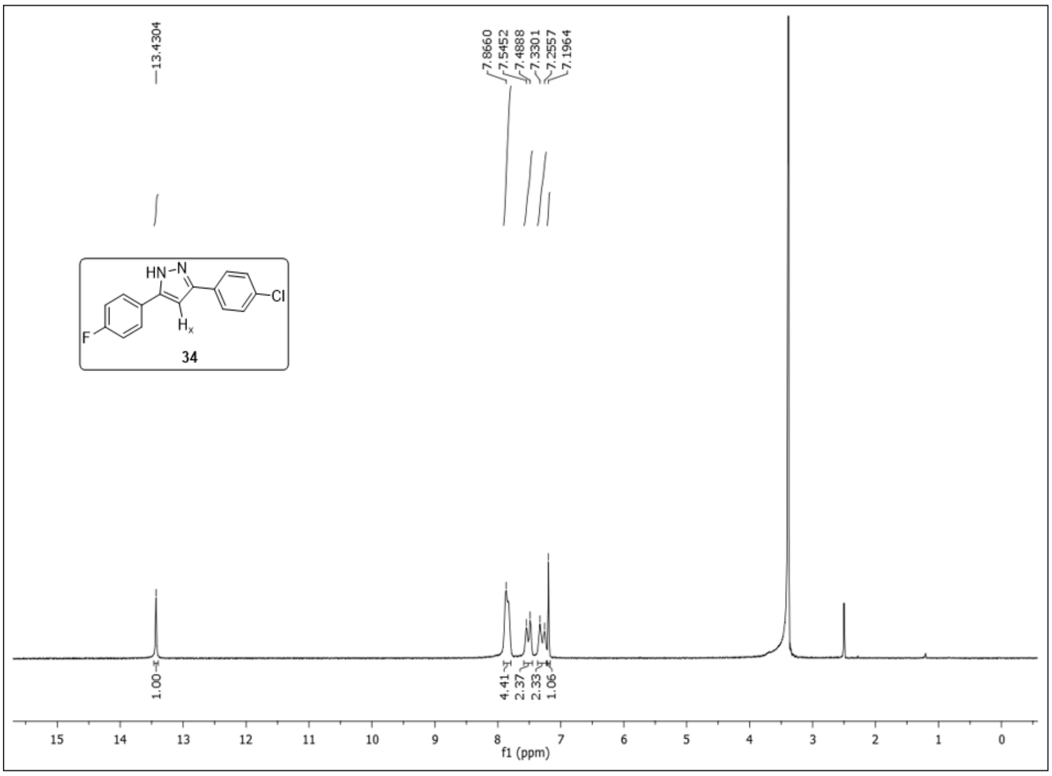

Figure S20. ^{1}H NMR of compound **34**.
